# Serious game for radiotherapy training

**DOI:** 10.1186/s12909-024-05430-1

**Published:** 2024-04-26

**Authors:** Jessika El Kayed, Tony Akl, Chadi Massoud, Christelle Margossian, Hadi Fayad, Georges Fares, Tony Felefly, Sandy Rihana

**Affiliations:** 1 Biomedical Engineering Department, Holy Spirit University of Kaslik, USEK, Jounieh, Lebanon; 2 Faculty of Public Health, Université La Sagesse, Furn-El-Chebak, Lebanon; 3 LATIM, UBO, Bretagne, France; 4 Radiation Oncology Department, Hôtel Dieu de France Hospital, Saint Joseph University, Beirut, Lebanon; 5 Radiation Oncology Department, Mount Lebanon Hospital, Hazmieh, Lebanon

**Keywords:** Serious games, Radiotherapy, Digital health, Medical education, VMAT - Volumetric Modulated Arc Therapy

## Abstract

**Background:**

Cancer patients are often treated with radiation, therefore increasing their exposure to high energy emissions. In such cases, medical errors may be threatening or fatal, inducing the need to innovate new methods for maximum reduction of irreversible events. Training is an efficient and methodical tool to subject professionals to the real world and heavily educate them on how to perform with minimal errors. An evolving technique for this is Serious Gaming that can fulfill this purpose, especially with the rise of COVID-19 and the shift to the online world, by realistic and visual simulations built to present engaging scenarios. This paper presents the first Serious Game for Lung Cancer Radiotherapy training that embodies Biomedical Engineering principles and clinical experience to create a realistic and precise platform for coherent training.

**Methods:**

To develop the game, thorough 3D modeling, animation, and gaming fundamentals were utilized to represent the whole clinical process of treatment, along with the scores and progress of every player. The model’s goal is to output coherency and organization for students’ ease of use and progress tracking, and to provide a beneficial educational experience supplementary to the users’ training. It aims to also expand their knowledge and use of skills in critical cases where they must perform crucial decision-making and procedures on patients of different cases.

**Results:**

At the end of this research, one of the accomplished goals consists of building a realistic model of the different equipment and tools accompanied with the radiotherapy process received by the patient on Maya 2018, including the true beam table, gantry, X-ray tube, CT Scanner, and so on. The serious game itself was then implemented on Unity Scenes with the built models to create a gamified authentic environment that incorporates the 5 main series of steps; Screening, Contouring, External Beam Planning, Plan Evaluation, Treatment, to simulate the practical workflow of an actual Oncology treatment delivery for lung cancer patients.

**Conclusion:**

This serious game provides an educational and empirical space for training and practice that can be used by students, trainees, and professionals to expand their knowledge and skills in the aim of reducing potential errors.

**Supplementary Information:**

The online version contains supplementary material available at 10.1186/s12909-024-05430-1.

## Background

Serious Gaming, as its name suggests, is the implementation of game principles and ideologies for serious matters in the real world such as education, engineering, or healthcare purposes. This idea first came to life in the 1970’s and was later defined by Stokes to be “games that are designed to entertain players as they educate, train, or change behavior.” In simple terms, they are video games mimicking real-life scenarios that take the user through different steps, activities, and testing to achieve an end goal or outcome, depending on the target of the game. Therefore, we can deduce that they are virtual platforms providing a realistic environment for users to benefit from by fulfilling the purpose of creating an entertaining learning experience [1]. Serious Games have been around for decades effectuating numerous objectives such as education, healthcare, defense, training, and many more [2–6]. Distinctively for healthcare, they are categorized into health monitoring, anomaly detection, therapy, education, and rehabilitation [7]. Even though its serious gaming domain is booming, it is still not as extensive as needed, noting its wide range of benefits highlighted throughout this paper which can potentially elevate the level of training in medicine. Regardless, they have a multitude of current applications; surgical procedures such as knee replacements and blood management, odontology for dentistry risk management, nursing training for patient pain regulation, cardiology for stroke rehabilitation assistance, first aid training, and the list goes on [3–6, 8]. There currently exists games including EMSAVE, OLIVE, PULSE!!, VIRTUAL PAIN MANAGER, and VIRTUAL ECG, that target, respectively, urgent medical procedures for the disabled, hospital training, clinical skills enhancement with patients of trauma [7], patient pain regulation for nurses, and ECG recordings precision for cardiology professionals [8].

With the current Covid-19 pandemic consuming today’s world, it established global challenges and induced the need to adopt new routines, habits, and measures as precautionary elements. We observed the rise of telemetry and e-Health to cut contacts with the virus, as well as e-Learning. Students suffered internationally from these limitations and drove them to avoid contamination by finding new methods of education and training. The pandemic therefore accelerated the world of digital education, specifically in the medical field, which is where the role of innovating computer-based platforms comes into play. In many areas, the radiology and oncology department adapted to this concept as well and began performing their radiotherapy planning in distance. However, the sudden shift to working remotely presented risks that could potentially affect the medical errors and their frequency [9]. It is important to note that on-site work is considerably different than standard theory in medicine since the presence of patients and first-hand examinations highlight the diversity of solutions and personalization, even for two patients of the same case. Consequently, Serious Games issue fundamental potential for this matter. Even when then need for isolation is no longer needed, the shift to the remote world is observably dominating that Serious Games can remain a powerful in the domain of learning and training, which can be utilized as an additional tool for students and professionals rather than be the sole source of knowledge.

Serious Games present a major tool for additional e-Learning purposes and encourage education and knowledge delivery by providing the sense of enjoyment and reward, with the added benefit of retaining information [10]. When effective, these games play a vital role in influencing players’ mood and expand their interest to proceed with the game and accomplish its goals. They are also proven to ameliorate the player’s cognitive skills and enrich their multidisciplinary endeavors and motivation [11]. When it comes to healthcare, training is a crucial part for professionals to go through and must be continuous over the years. However, training has evolved to keep up with medicine over time until Serious Gaming.

Our target in this project is to develop an educational tool for medical physicists, radiologists, and students to immerse them into the radiology process steps, and precisely related to Lung Cancer patients. In such treatments, patients are exposed to emission energies ranging from 6 to 16 MV and radiation of 54–74 Gy thus inducing a limited margin of error [12]. Therefore, this paper covers our project presenting the first Serious Game for Radiotherapy Training including the entire clinical process, testing the player’s knowledge and decision-making skills, and reducing the alarming and life-threatening errors that may occur through a series of carefully studied and practical steps.

## Materials and methods

To design a serious game that illustrates the real world of radiotherapy treatment, a tangible exploration of a radiotherapy department is necessary. This project has been done in collaboration with LaTIM laboratory in Brest, France, and Mount-Lebanon Hospital in Beirut, Lebanon, who provided the required clinical information to build and validate the game.

### Educational framework and objectives

The serious game is structured around key stages of radiotherapy, each designed to target specific educational objectives aligned with essential competencies in radiotherapy. The aim is to provide a holistic and interactive learning experience, blending clinical accuracy with engaging, scenario-based tasks.

### Clinical radiotherapy procedure, game stages and learning outcomes

The radiotherapy treatment plan consists of the different steps: Screening, Contouring, External Beam Planning, Plan Evaluation, and Treatment Delivery.

#### Screening stage

##### Objective

To enhance understanding of patient consultation and tumor visualization techniques.

##### Interactive methods

This stage includes scenario-based learning where users make decisions on patient data and imaging techniques. Feedback is provided to reinforce correct practices and explain errors.

##### Procedure

The procedure first begins with the consultation of the lung cancer patient with a radio-oncologist who will select the treatment. He/she undergoes a CT scan to visualize and study the tumor, its size, and position. Besides, imaging is also used to locate and calculate its iso-center relative to that of the radiotherapy system. The oncologist will use this information and mark the contour of the tumor for the mobile laser beams to use as a center and mark as tattoos. The patient must be stable during the imaging using stabilizers that hold the arms and legs to reduce mobility and attenuation caused by the bones. In this case, the Maximum Intensity Projection (MIP) treatment method can be applied which takes at least 3 scans in different breathing states to obtain the average intensity of the images and compute the persisting location of the tumor. There is also the Respiratory Gating treatment method that incorporates a Varian Real-time Position Management system to perceive the breathing of the patient during continuous CT acquiring. However, this method is more irradiating, therefore used the least [13].

The previous data is utilized to plan the treatment of the patient using a Treatment Planning System, which is the Eclipse model created by Varian at LaTIM made strictly for research.

#### Contouring stage

##### Objective

To develop skills in delimiting target volumes and organs at risk.

##### Interactive methods

Users engage in drag-and-drop contouring exercises, with immediate feedback on accuracy. This stage is crucial for understanding spatial relationships in radiotherapy planning.

##### Procedure

Contouring is performed to delimit, and reconstruct the organs at risk and the target volumes that consist of Gross Target Volume (GTV) which must acquire all the prescribed dose, the Clinical Target Volume (CTV) which considers the tumor extensions, and the Planning Target Volume which considers internal tumor mobility of the cancer. It is rather crucial to avoid the organs at risk and compute the radiation received by them, and with lung cancer being our study, the OAR are the heart, lungs, esophagus, and spinal cord. Note that it is not enough to contour these specific areas, but this must also be computed for the whole body and the scanner table to measure the attenuation of the beam. This is possible within the software due to the contouring tools used by the radiologists [13].

#### External Beam Planning

##### Objective

To impart knowledge on selecting and creating appropriate radiation treatment plans.

##### Interactive methods

The game presents various patient scenarios, challenging the user to choose and customize treatment plans using interactive tools, followed by quizzes to test their understanding.

##### Procedure

After the calculations have been completed and contouring is made, beam planning can begin. This step is the process of creating and selecting the best program depending on the patient’s case and his/her specific medical needs. There exist three main types of programs being Three-dimensional conformal radiation therapy (3D-CRT), Intensity-Modulated Radiation Therapy (IMRT), and Volumetric-Modulated Arc Therapy (VMAT) [13]. These treatment plans can be compared, and the one that fits the patient best is chosen to be evaluated and validated.

#### Plan evaluation stage

##### Objective

To train users in assessing and validating treatment plans.

##### Interactive methods

This stage utilizes case studies and comparative analysis exercises, enabling users to evaluate different treatment plans based on dosimetric and clinical criteria.

##### Procedure

After the calculations have been completed and contouring is made, beam planning can begin. This step is the process of creating and selecting the best program depending on the patient’s case and his/her specific medical needs. There exist three main types of programs being Three-dimensional conformal radiation therapy (3D-CRT), Intensity-Modulated Radiation Therapy (IMRT), and Volumetric-Modulated Arc Therapy (VMAT) [13]. These treatment plans can be compared, and the one that fits the patient best is chosen to be evaluated and validated.

##### 3D-CRT Planning procedure

This conformal planning utilizes 6 beams of photon radiation penetrated at multiple angles to reduce its exposure to the organs at risk and affect healthy tissue. We can form this plan in the planning software where we input the CTV, required dose, and aim of treatment. Additionally, we manually alter the angle of rotation, add the beams with an energy of 6x, and add a Multi-Leaf Collimator (MLC) at every beam. The dose must be carefully selected as the maximum value per fraction and the number of fractions to be undergone, and the computational algorithms are to be selected as the calculation model. It is also feasible to regulate the Dose-Volume Histogram (DVH) which designates the percentage of radiation each volume is exposed to. It can also be a guide for plan evaluation and comparison. Afterwards, the plan undergoes an evaluation to obtain a validation and it is simulated on a Phantom to verify the ability of the system to deliver the same treatment [13].

##### IMRT Planning procedure

This radiation therapy follows the same steps as the conformal planning, but the major difference is that at the MLC addition step, they are added automatically rather than manually. It’s important to note that this automation of MLC is not exclusive to Intensity-Modulated Radiation Therapy (IMRT) but is also a feature of other techniques such as Dynamic Conformal Arcs (DCA), which, despite utilizing automated MLC, do not fall under the category of IMRT.

The system will generate the optimum solution overcomes the dosimetrist’s constraints and simulates the opening of the MLC to deliver the ideal dose to the tumor while minimizing it to the adjacent tissues [13].

##### VMAT Planning procedure

**Fig. 1:**
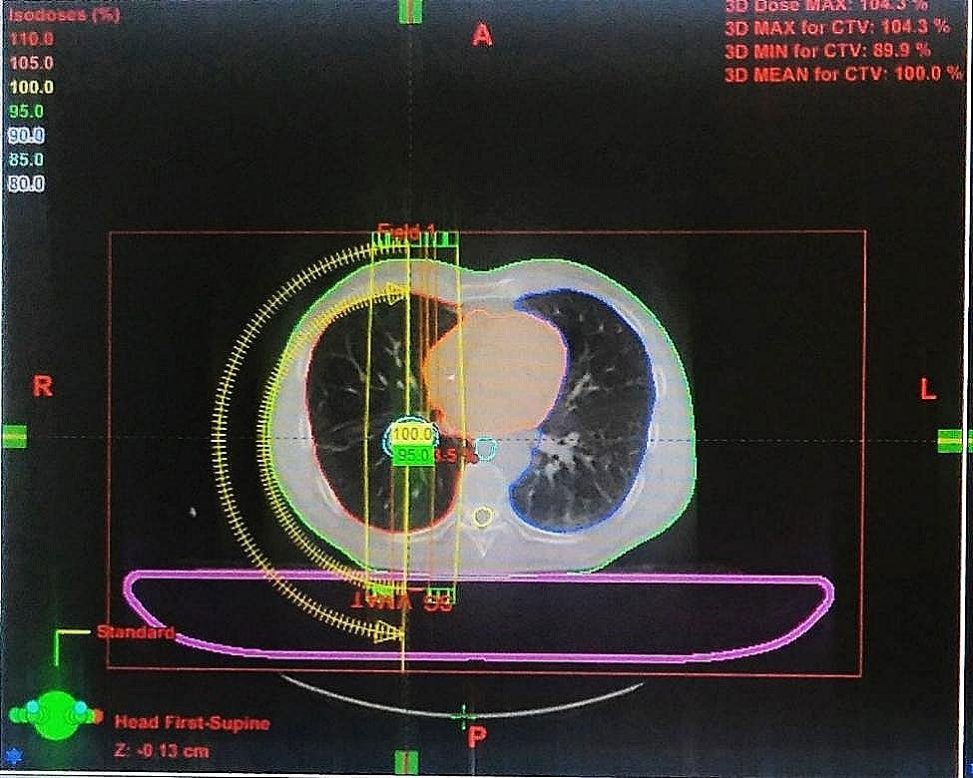
VMAT Arcs

VMAT is established on the same planning type as IMRT. However, the treatment is based on an arc and the Multi-Leaf Collimator is set to switch positions at each of its rotations. We can optimize it by picking the number of isocenters and half rotations, being 1 isocenter and 2 half rotations in our case, meaning two treatment arcs rotating at 180˚ deliver the radiation [13]. The system will spread the dose of radiation over the whole volume as see in Fig. 1.

**Fig. 2:**
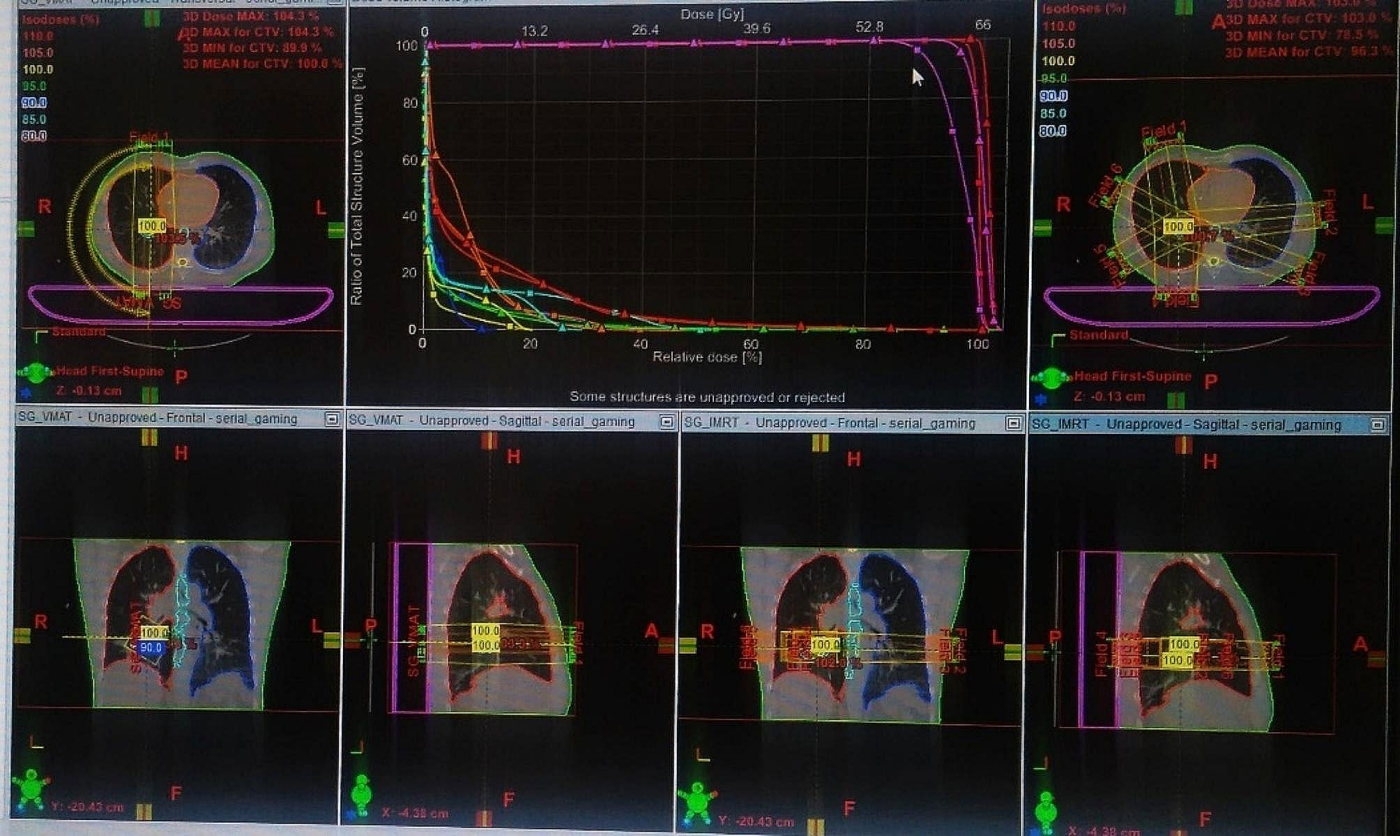
Comparison of Plans (VMAT et IMRT)

We can also compare between the two planned treatments (IMRT and VMAT for example) to choose the best option for the patient. Thus, the best choice will be validated and simulated to be delivered to the patient Fig. 2.

#### Treatment delivery stage

##### Objective

To simulate the process of administering radiation therapy accurately and safely.

##### Interactive methods

The game incorporates a virtual radiotherapy suite where users position patients and deliver prescribed treatments, receiving real-time feedback on their precision and adherence to safety protocols.

##### Procedure

**Fig. 3:**
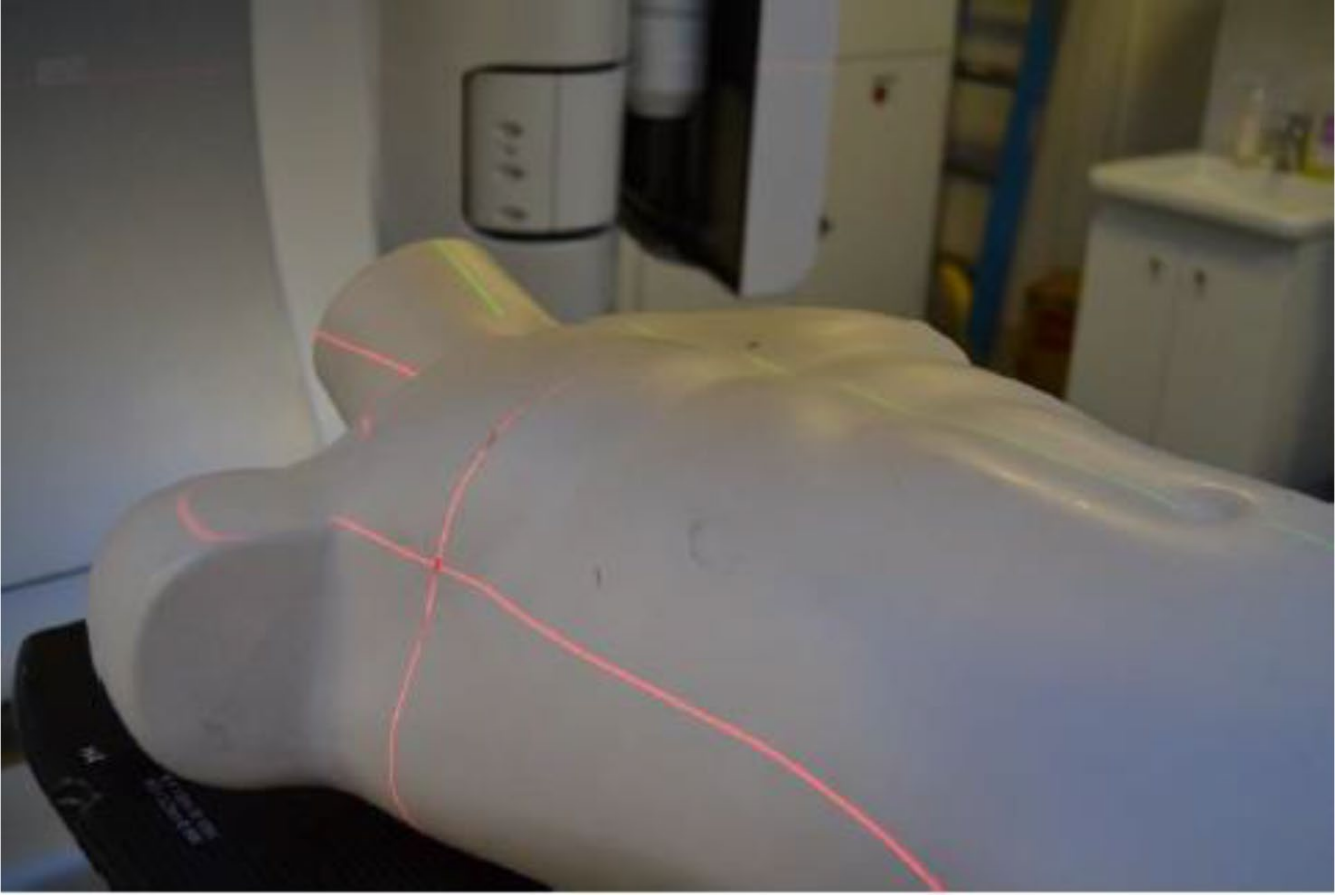
The mannequin’s placement is crucial to adjust the corresponding CT markings and ensure the the exact positioning before irradiation begins

The patient will now undergo a carefully calculated and personalized treatment of 66 Gy in 33 fractions of 2 Gy each in the Radiotherapy room to limit the amount of radiation exposure per session. Note that the dose and fractionation numbers may vary depending on several factors such as the computations, the case of the patient, the length of treatment, the tissue response to radiation, and the tumor radiosensitivity [14]. The patient lays based on the tattoos resulting of the CT scan and the isocenter of the machine detected by a laser system. X-rays must be captured to compare with the CT scan to guarantee correct positioning of the patient with respect to the CT markings as seen in Fig. 3; this is then when irradiation begins. One CBCT image is taken every week for reference.

### Game Design and Development

The game was developed in collaboration with clinical experts to ensure clinical relevance and accuracy. State-of-the-art 3D modeling [15] and animation techniques were used to create realistic and immersive game environments.

#### Real case shadowing

A real-life case of a Stage III NSCLC male patient who was given a VMAT treatment with a dose of 60 Gy separated over 30 fractions has been shadowed at the Mount Lebanon Hospital in Lebanon. The steps taken consisted of delineating the areas of the CT scan, performing contouring of the multiple volumes and organs at risk, and executing the external beam planning, the 3D-CRT in specific. This plan was then compared with the original VMAT plan. After plan evaluation, the treatment resulted with a well-distributed radiation and no hot spots. It then undergoes a plan validation through a verification plan as a Quality Assurance tool to be then delivered to the patient.

#### Gamification

Integrating the radiotherapy procedure within a game can be challenging; this is due to trying to transform a critical procedure into a set of interactive game elements with the use of different software [16]. These elements formed based on the “Octalysis Gamification Framework” [17] that relies on multiple cores which focus on merging the player’s emotions and motivation to succeed in the game in an iterative manner while learning. It is designed to stimulate different parts of the brain for optimum results.

#### Implementation of interactive methods

Throughout the game, interactive methods such as multiple-choice quizzes, drag-and-drop activities, and scenario-based decision-making are employed to ensure active learning and retention of information. These methods are designed not only to test knowledge but also to provide immediate feedback, thereby reinforcing learning and correcting misconceptions.

### Tools and platforms

#### Unity Game Engine

Unity is a game engine platform that includes multiple features to establish a realistic medical environment for radiotherapy [18]. It includes the physical calculations and graphical modeling of the 3D objects we want to create [19–21].

Scripting is a crucial part of every game object that is useful to control and manipulate them, enabling and disabling features, playing animations, etc. They are coded using an appropriate language, being C# in this project.

#### Autodesk Maya 2018

To bring our model into existence in the game, Autodesk Maya 2018 is a useful software to create the models and make them more realistic by customizing the materials and textures, rigging, and animating them in a controllable manner. After completion, we should export our work to our game engine and finally design the game.

## Results

### Modeling and Animation

**Fig. 4:**
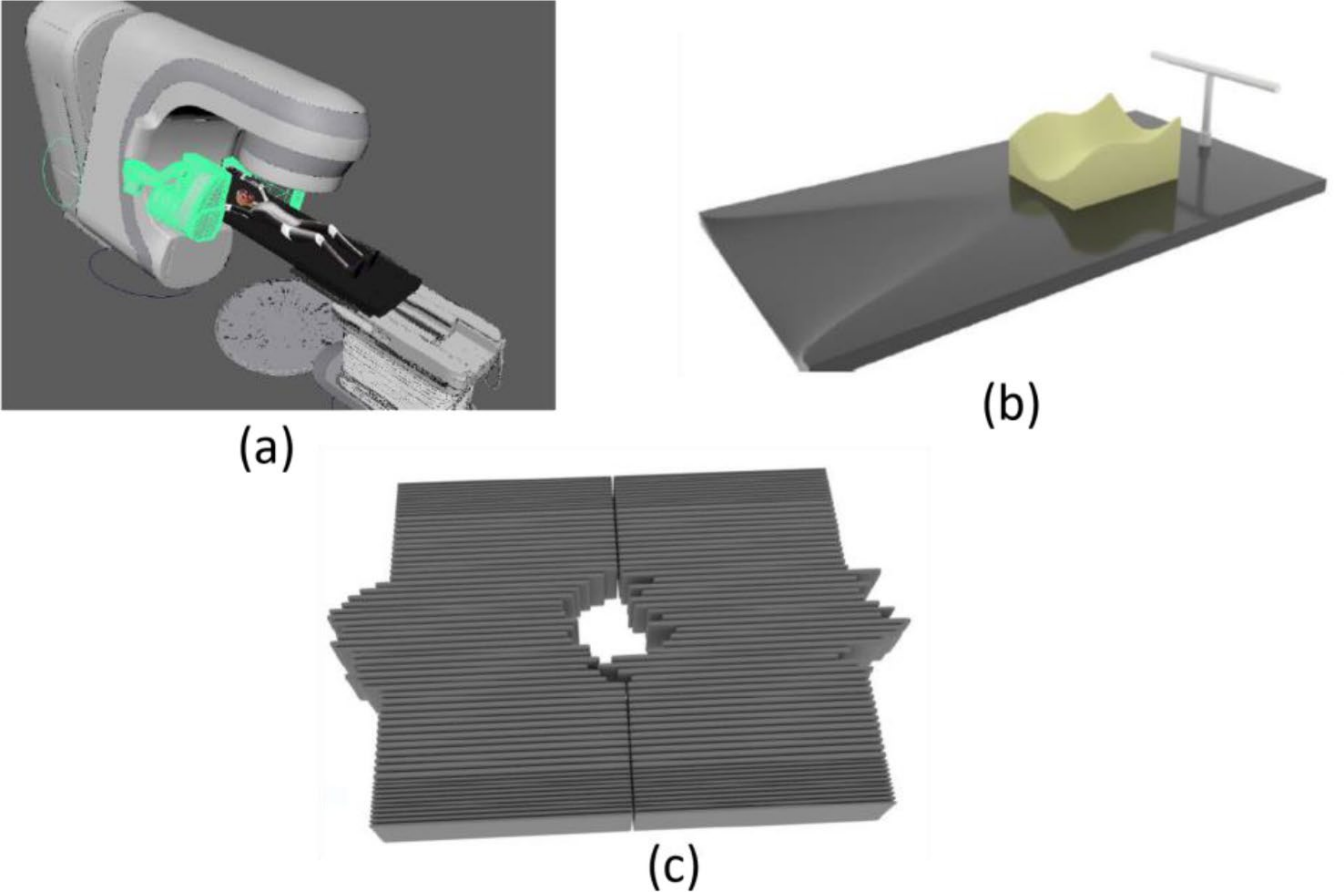
(**a**) The animation of the Truebeam kV Control Curve altering the detector positioning of the beams. (**b**) The animation of the Thorax Immobilizer was designed on Maya 2018 Render with a smoothened headrest and a T shape consisting of 2 cylinders for the patient to hold on to. (**c**) The 120-leaves Multi-Leaf Collimator shown was modeled on Maya 2018 Render and rigged to simulate the three types of treatment; one attribute for 3D-CRT and another for IMRT and VMAT

To accomplish our project, the animated models were merged into realistic scenes. Figure 4 (a) illustrates a standard radiotherapy model, with materials and textures integrated onto it where a control curve is created for the kV source and detector movement.

Figure 4 (b) portrays the thorax immobilizer that was modeled along with a headrest for the patient. Furthermore, the 120-leaves Muti-Leaf Collimator has been modeled as seen in Fig. 4 (c) with a rough metallic material for a more realistic look. Most importantly, the model was rigged by forming an attribute for the 3D-CRT, and one of the IMRT and VMAT treatments.

### Unity scenes

**Fig. 5:**
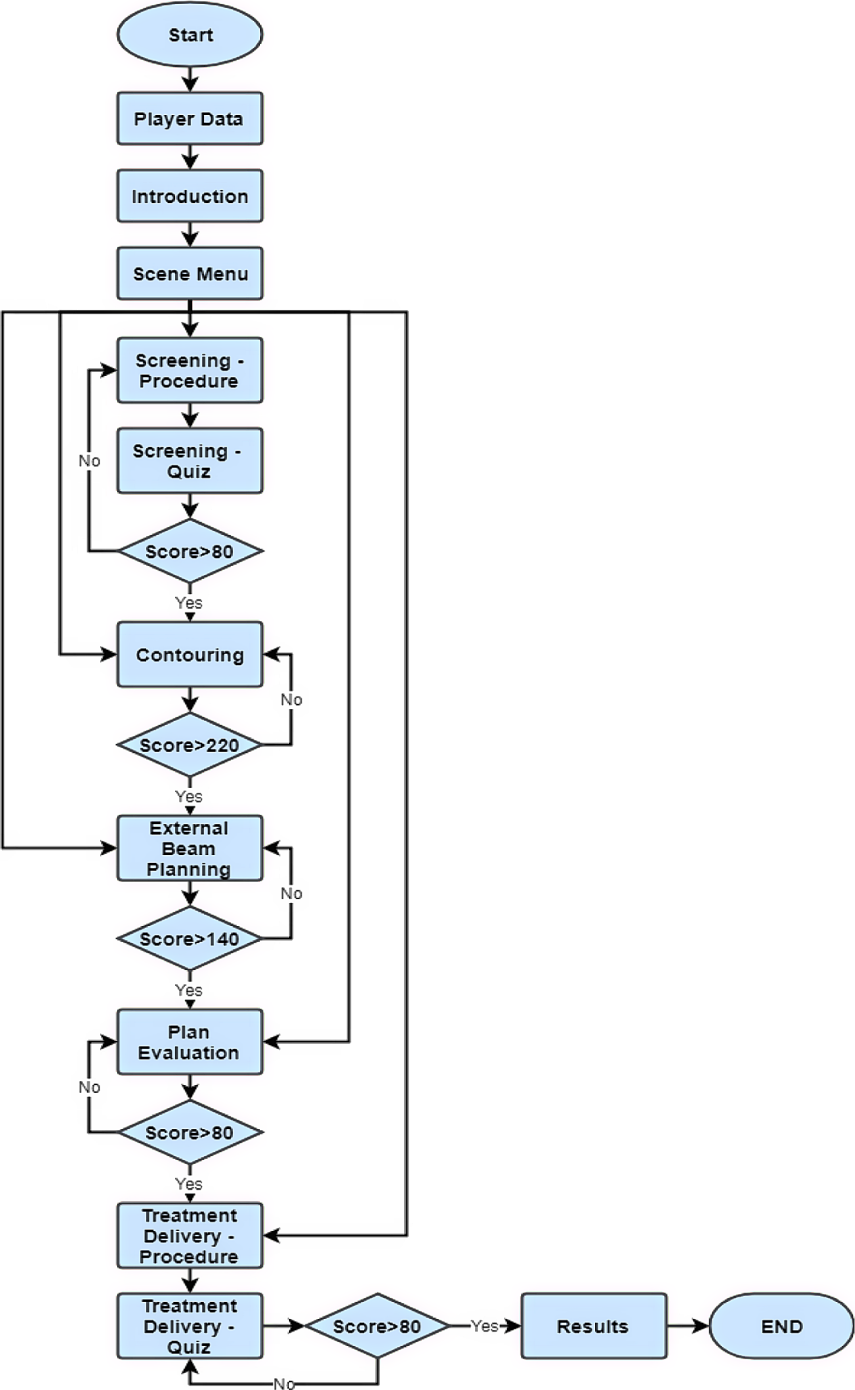
This is the Serious Game Flowchart from Start to End. The player inserts his data, then goes through the different clinical steps of radiotherapy treatment through which he/she can navigate if the score is above a certain number. The results are then displayed and the game ends

To accomplish the formation of a training tool, we created our Serious Game to test the player’s skills and knowledge. It goes through the different steps of the radiotherapy procedure and a respective scene for each of 5 steps. Figure 5 represents the flowchart of the game from start to finish. The player must exceed a certain set score to move to the next step. Quizzes consisting of multiple choice, True/False, and Drop and picture selection questions are also incorporated into the flowchart to test the player’s information and provide additional facts and comments within. Continuous assessment is crucial in the learning process of the player and provides a chance to apply the content in practice. Additionally, including a variety of testing methods is more engaging and entertaining to the player, especially since each method can induce a new way of thinking for the player’s analytical and decision-making skills. This can also enhance memory, aid in recall, and improve recognition [22].

Our game consists of 10 scenes, going from player data collection to scene selection and a results scene. It also simulates the five main phases of a radiotherapy treatment: Screening, Contouring, External Beam Planning, Plan Evaluation and Treatment Delivery. In addition, it tests the players’ knowledge using interactive quizzes, providing them and people in charge of their training with small analytics.

**Fig. 6:**
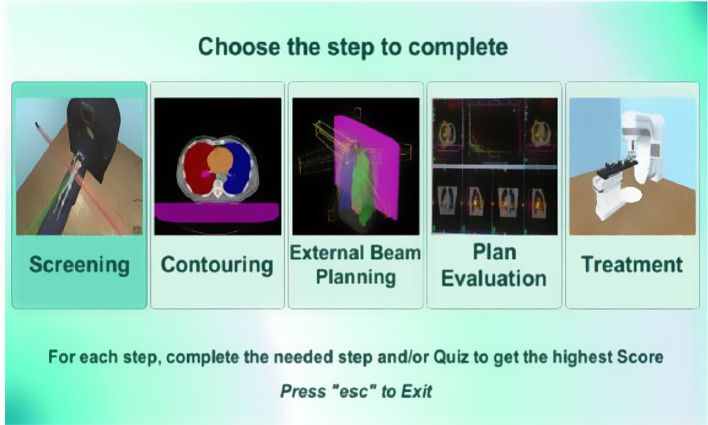
This is the Menu Scene displayed after the uses enter their data. İt shows all the radiotherapy treatment steps for the user to choose from, complete, and continue the game

The first scene of the game displays input fields to insert the player’s information. It is then followed by a Menu Scene seen in Fig. 6 that includes the different steps of the radiotherapy procedure; the player can begin from the phase they desire and move on to the next.

**Fig. 7:**
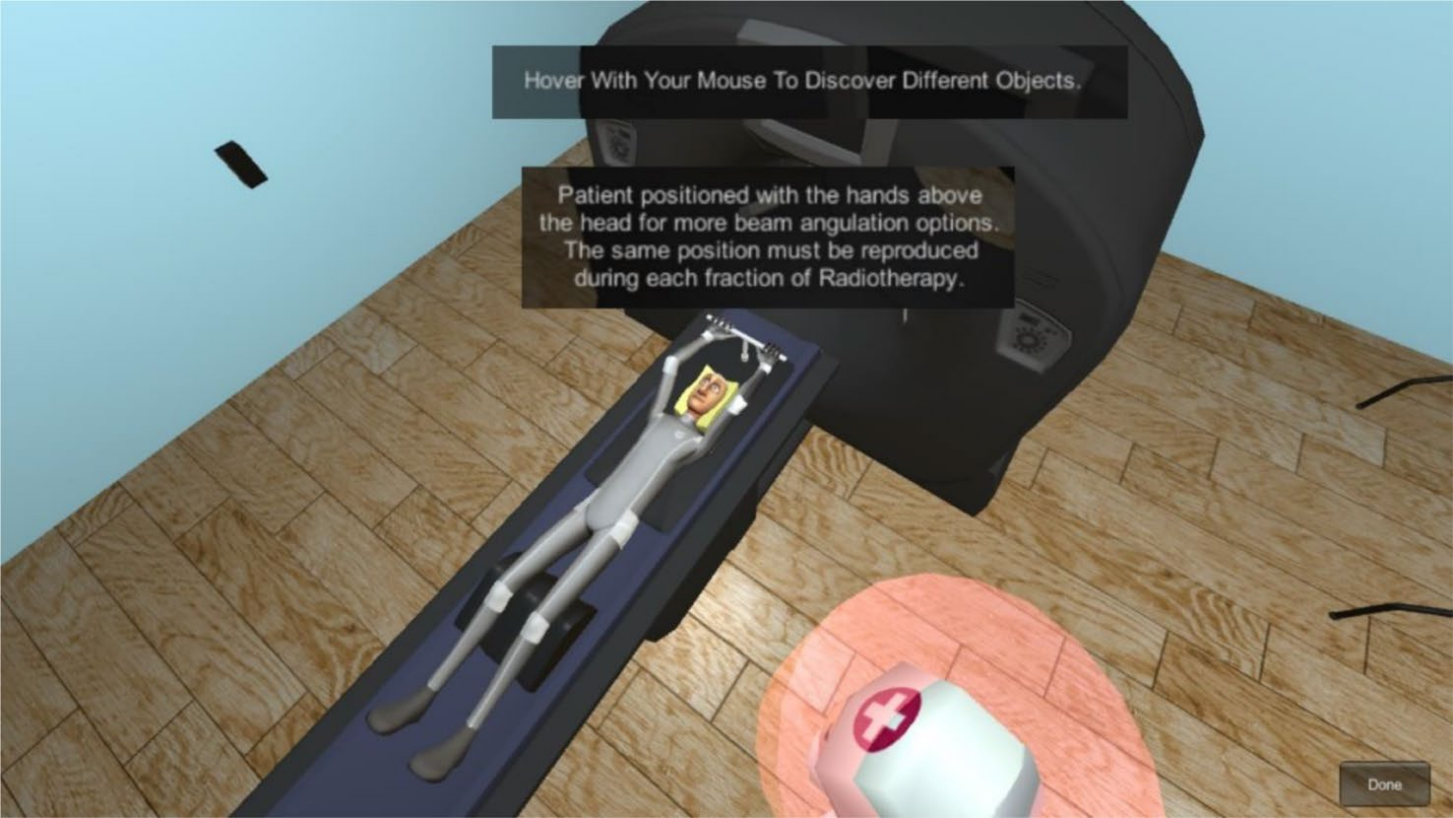
Screening Simulation Scene - Patient Positioning - The player can explore how the patient is positioned and why this position should be reproduced

Each scene has its medical environment for every step, and its respective flowchart to guide the player. Figure 7 represents an example of the realistic screening simulation scene, which is the first step of the radiotherapy clinical process in the game. Two additional figures related to this scene can be found in additional file 1.

**Fig. 8:**
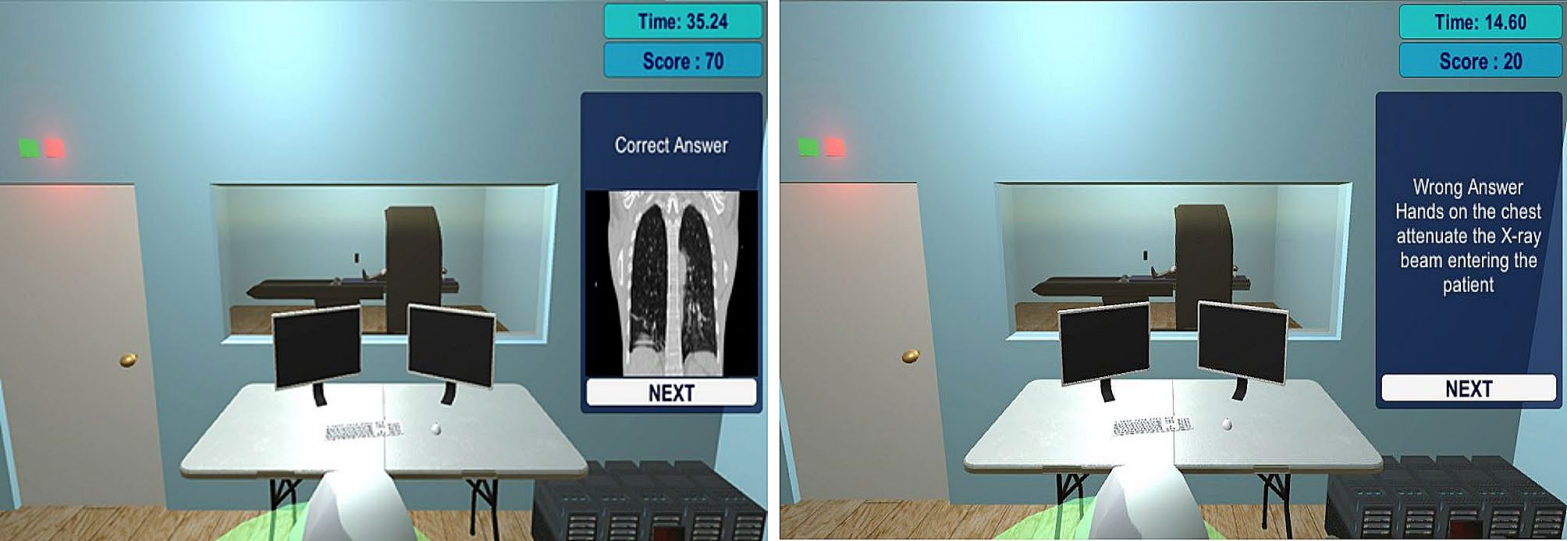
Screening Quiz Scene - Message Boxes - After each answered question, the player is given a message box showing whether he answered correctly or not, with additional information in some cases

According to the game flowchart, this is then followed by the Screening Quiz. The following image (Fig. 8) illustrate a screenshot example from this scene. One additional figure related to this scene can be found in additional file 2.

**Fig. 9:**
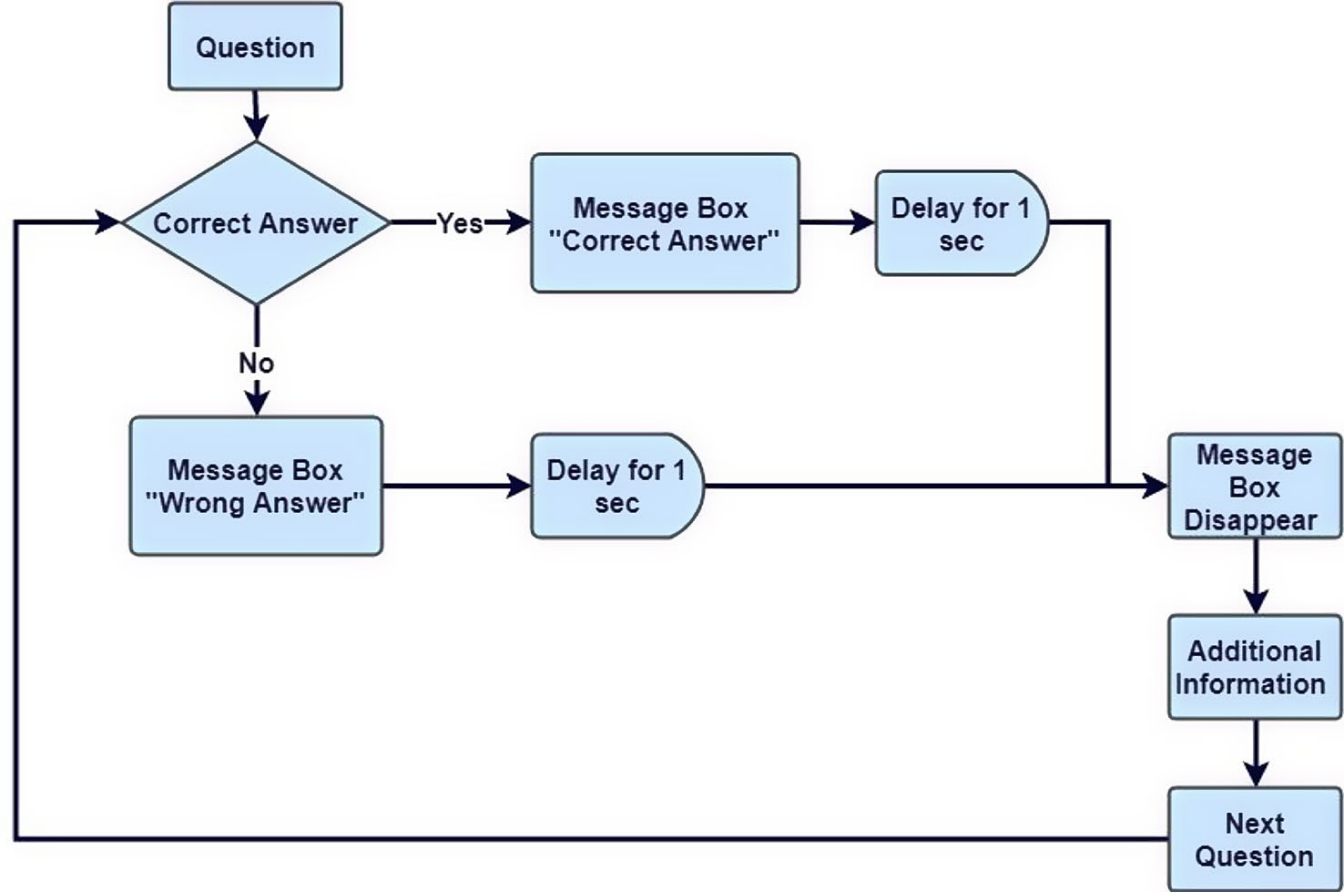
This is the flowchart of the Contouring Scene Questions made to test the player. As seen in the figure, whether the user answers correctly or not, additional information will be given to expand their knowledge and guide them

Then the Contouring Scene to test the player’s skills on delineating the patient’s target volumes and organs at risk, and knowledge through multiple choice, True/False, and Drop and picture selection questions. Figure 9 displays the flowchart of this scene and an example of our aim’s implementation into this game.

**Fig. 10:**
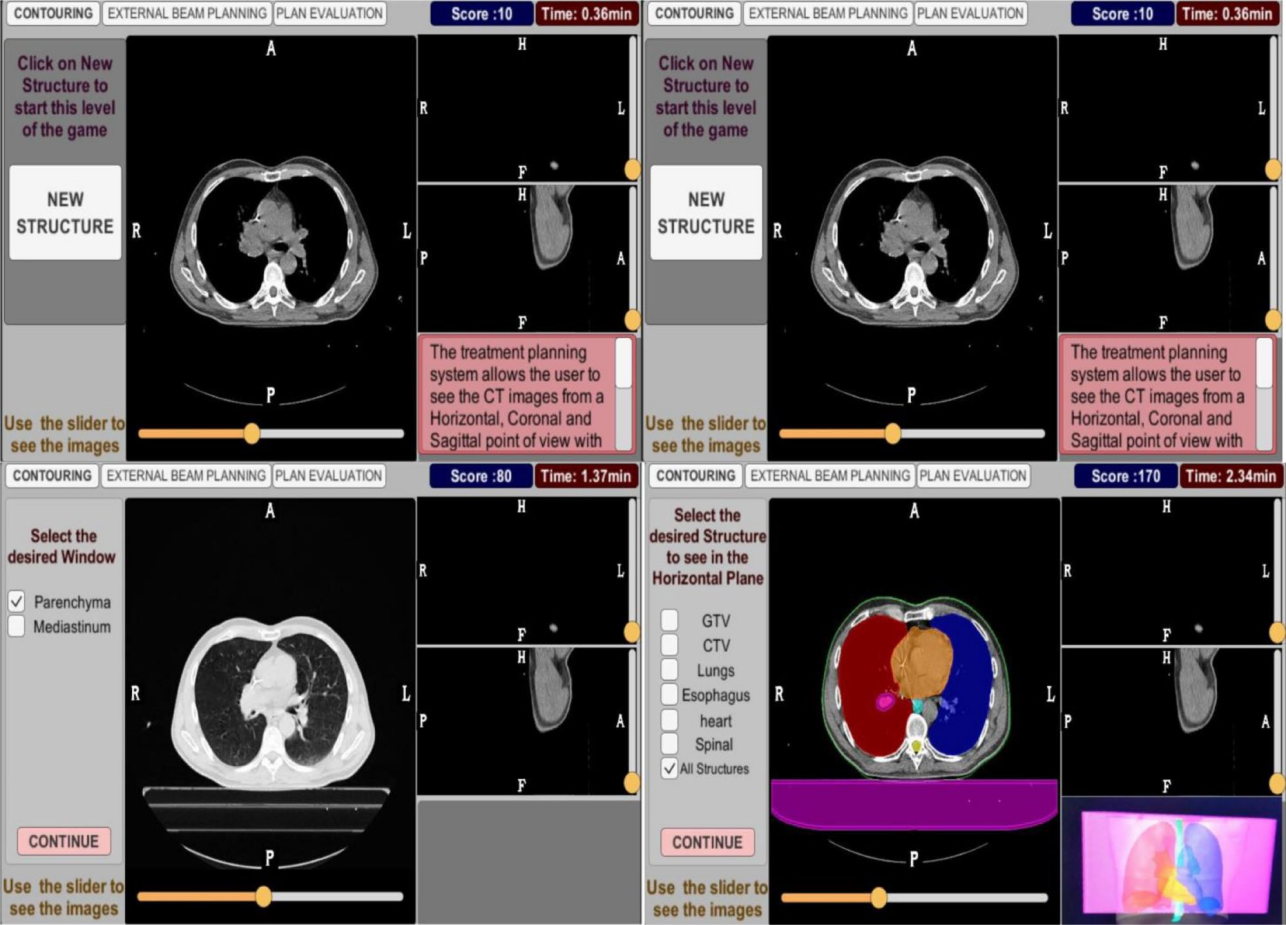
Contouring Scene - Clinical Images - Here, the player can explore loaded image datasets of a real patient, visualize different delineated structures such as target volumes and organs at risk, differentiate between windows. The player may use the sliders to scroll the dataset and select the structure to visualize on the left

Figure 10 illustrates a screenshot example from this scene. Three additional figures related to this scene can be found in additional file 3.

**Fig. 11:**
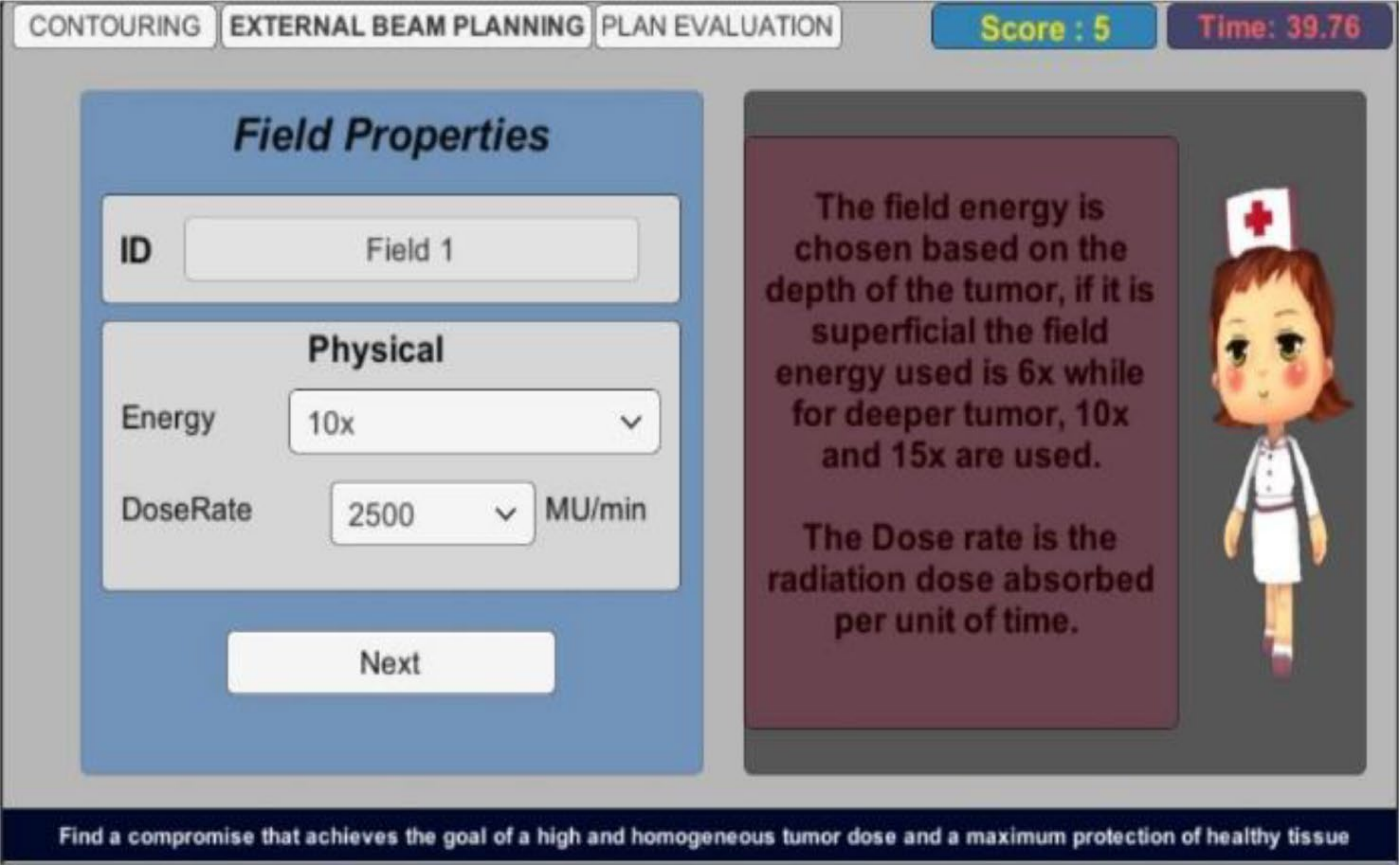
External Beam Planning Scene - Common Planning Steps - The common steps of the treatment planning are presented to the player with additional information explaining each step. Field properties include Energy and Dose Rate. Field energy is chosen based on the depth of the tumor. If it is superficial the field energy used is 6x while for deeper tumor, 10x and 15x are used. Dose rate is the radiation dose absorbed per unit of time

As for external beam planning scene, it incorporates a thorough explanation of the different types of planning and continues to test the player and his decision-making skills. Figure 11 illustrates a screenshot example from this scene. Two additional figures related to this scene can be found in additional file 4.

**Fig. 12:**
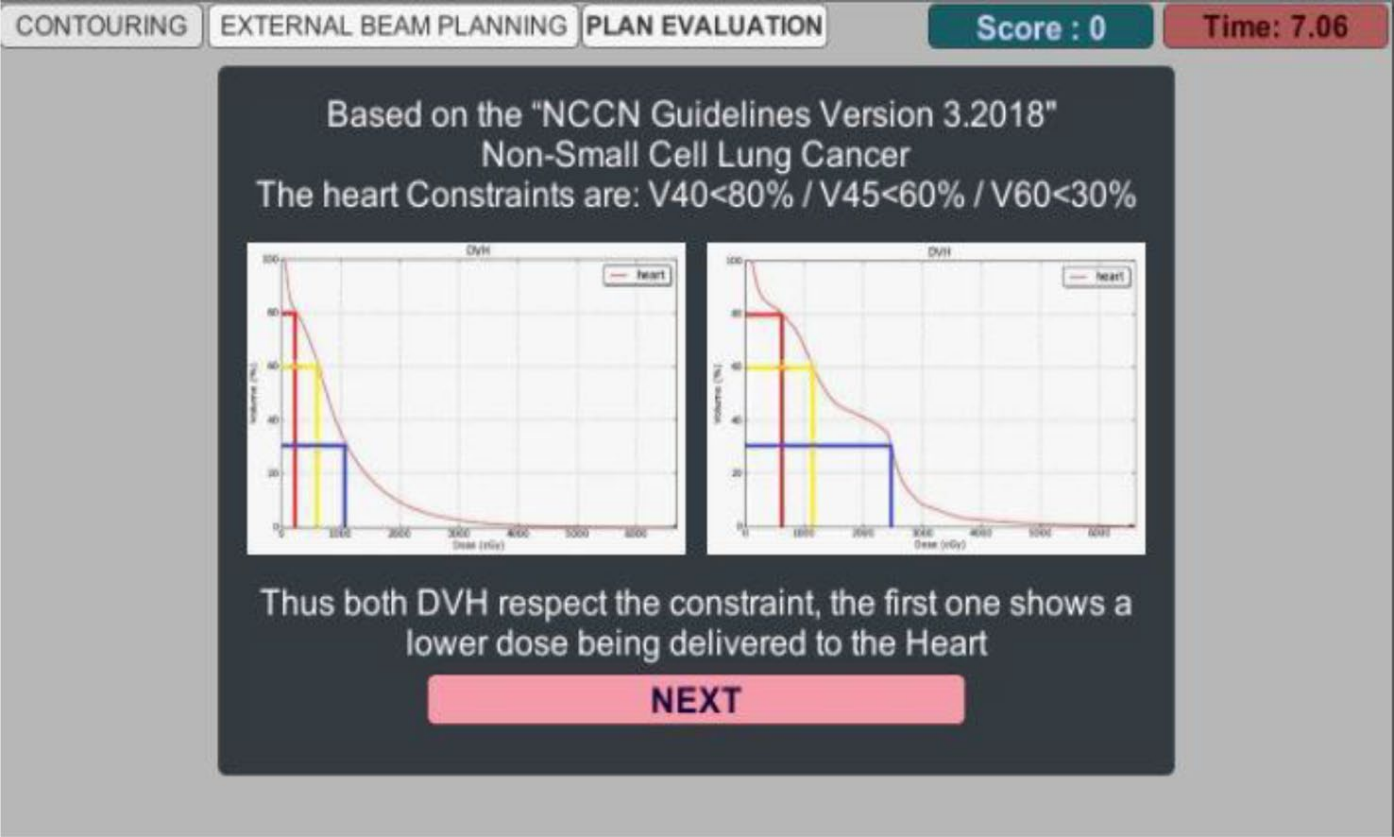
Plan Evaluation Scene - DVH Comparison - For the player to take the decision, the standards used are explained concerning the DVH and the iso-dose. Based on the “National Comprehensive Cancer Network” Guidelines for Non-Small Cell Lung Cancer, the heart constraints are V40 < 80%, meaning that 80% of the organ volume can receive up to 40 Gy, V45 < 60% and V60 < 30%. Two DVH results are presented, both respect the constraint, but the first one (on the left side) shows the lowest dose being delivered to the heart and by that it is the correct decision to make

Subsequently, the Plan Evaluation scene appears, providing a comparison tool for the player to assess the treatment plan according to iso-dose distribution and the DVH computed for each one. Figure 12 illustrates a screenshot example from this scene. One additional figure related to this scene can be found in additional file 5.

**Fig. 13:**
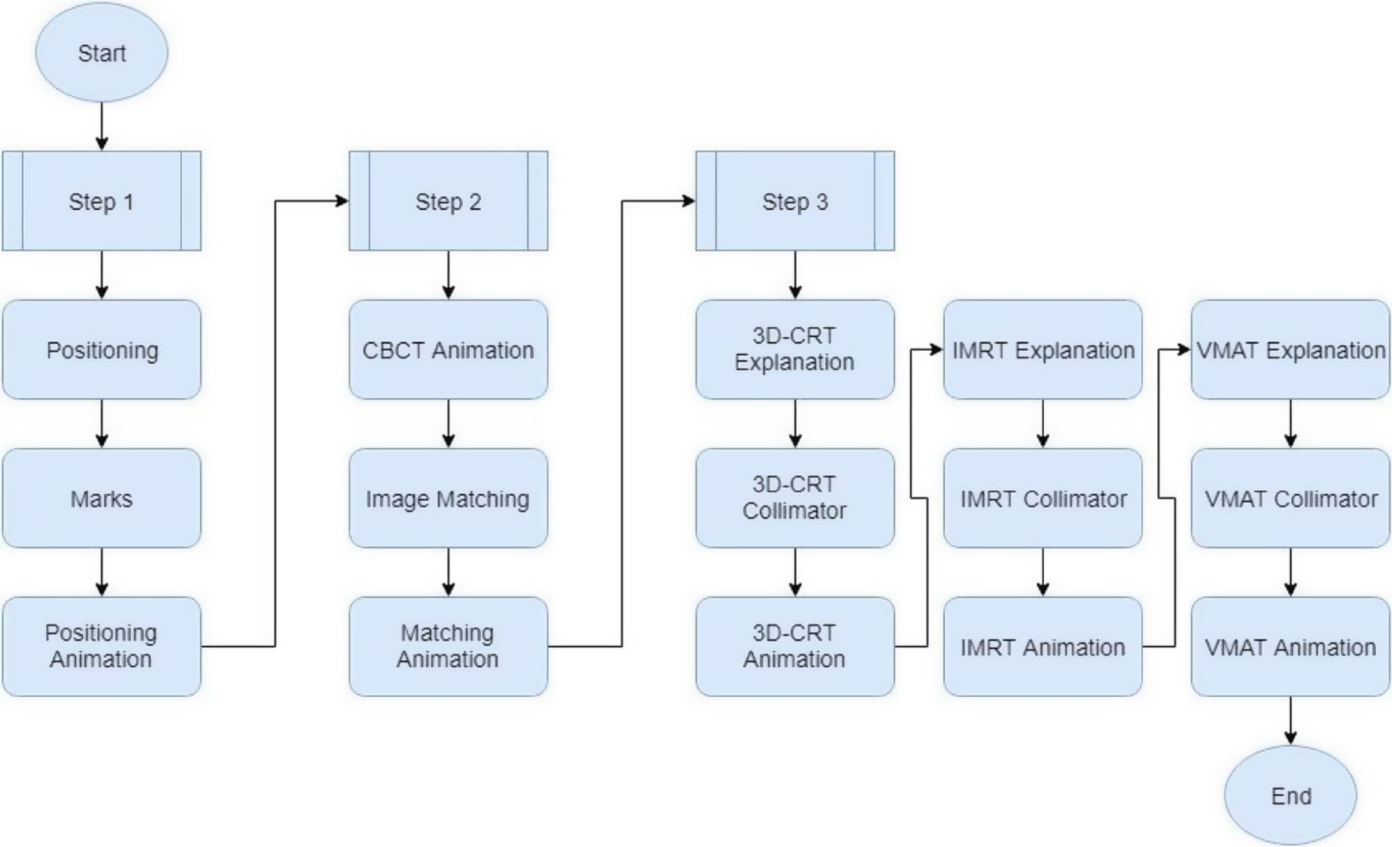
This is the flowchart for the Radiotherapy Treatment Delivery Scene the displays the steps embedded within the game to administer the treatment to the patient

**Fig. 14:**
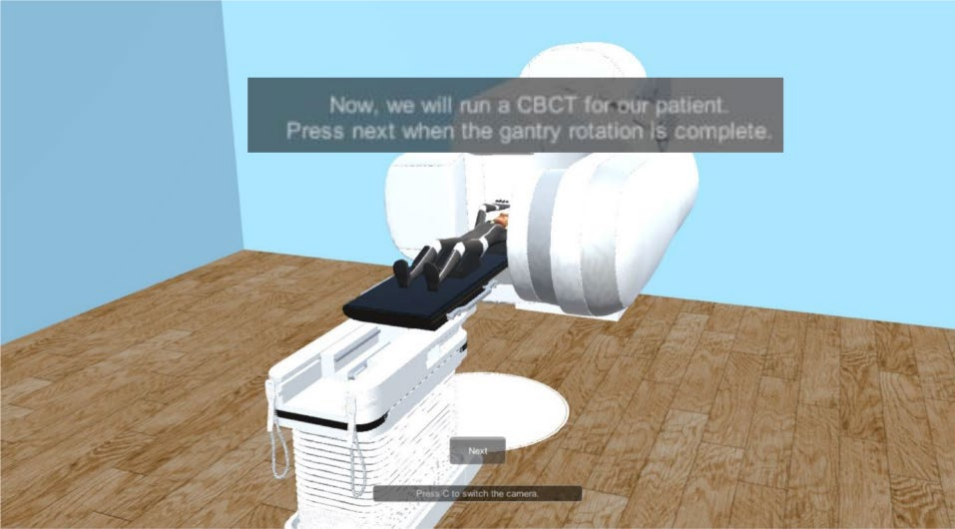
Treatment Delivery Simulation Scene - CBCT - The patient undergoes a CBCT on the first treatment, and once a week to validate the positioning of his tumor in the center of our Radiotherapy plan. Here, you may see the extended kV source (above the patient) and detector (under the table), taking images between 0˚ and 180˚ for CT reconstruction

**Fig. 15:**
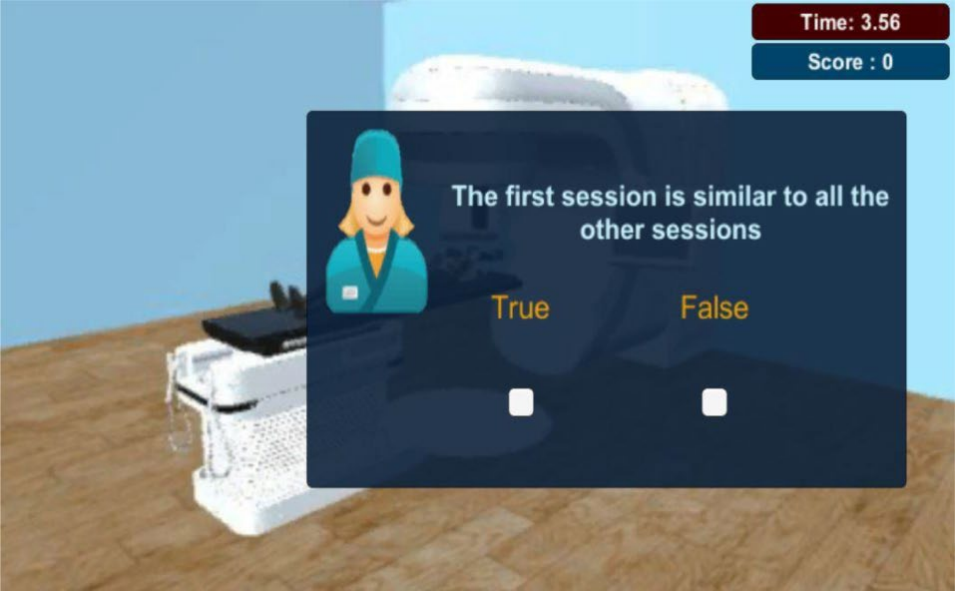
Treatment Delivery Quiz Scene - Questions - The first treatment session is not the same as the rest of the sessions. During this one, called J0, the patient position is adjusted and marked in order to be reproduced during the whole treatment

Finally, for the treatment delivery simulation scene, it begins with the correct positioning of the patient and delivering the respective treatment. As seen in Fig. 13, this flowchart describes the phases taken for the administration of the steps. Figure 14 illustrates one screenshot example from this scene. Knowingly, this scene is then followed by a final quiz. Figure 15 illustrates a screenshot example from this quiz scene. One additional figure related to this scene can be found in additional file 6.

**Fig. 16:**
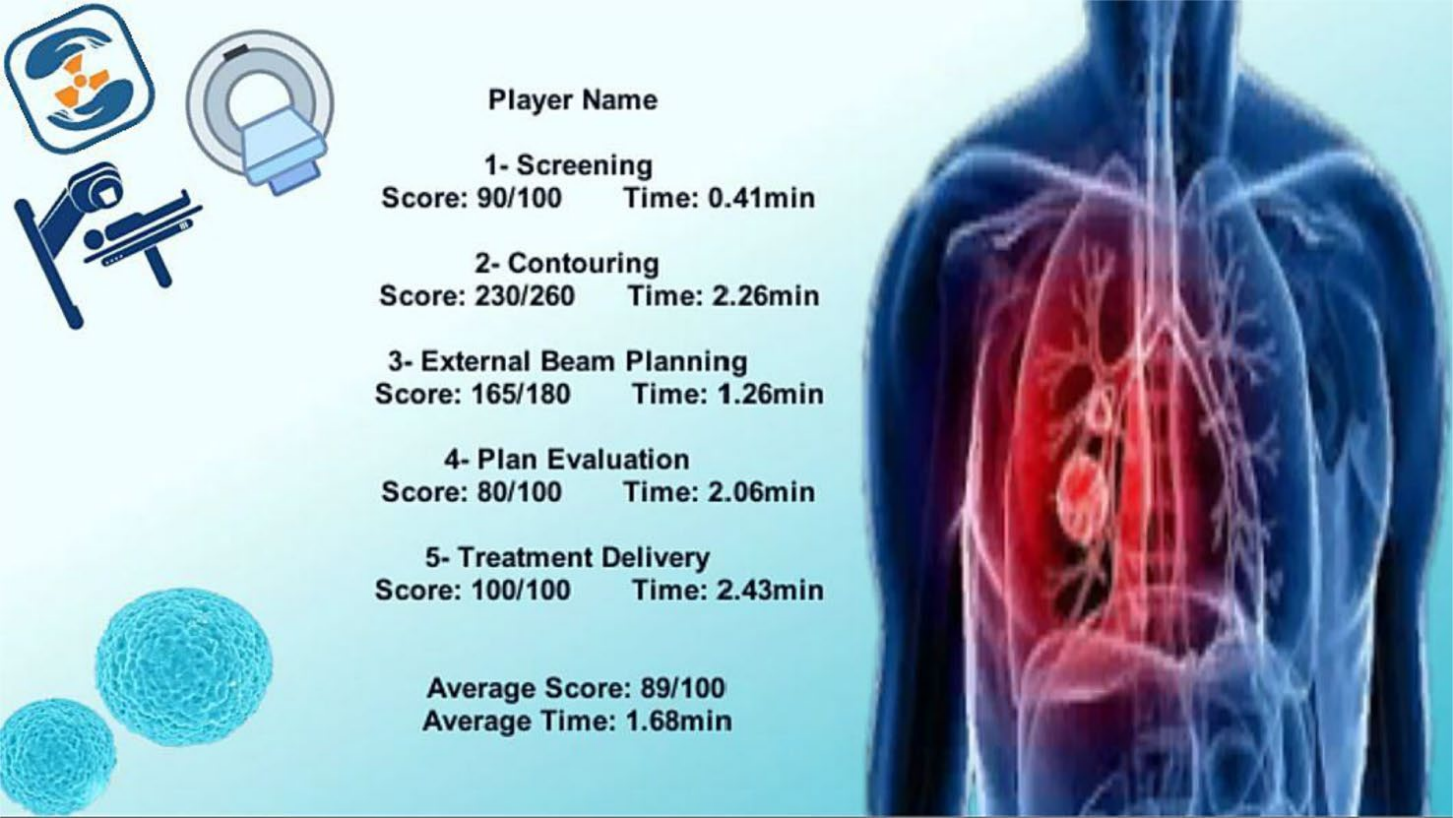
After the user completes all the steps, the Results Scene appears showing the summary of the player’s performance throughout the game

The final scene in the game is evidently the Results Scene as represented in Fig. 16 for the players and supervisors to review and is also proof of progress and time taken which are all stored within the game.

## Discussion

### Main Findings

Medicine is evolving at an expeditious pace, compelling humans to discover new ways to adapt to this rate. In addition to that, the rise of inevitable epidemics and pandemics taking over the world or parts of it, learning how to cope and protect ourselves is consequently essential. One of the biggest examples is the Covid-19 virus that overtook the population and disrupted our lifestyle and routine, and the best way to reduce contracting it is through social distancing and limiting excess interactions with others. Accordingly, professionals and students in the medical field had limitations concerning education, internships, and especially on-site training. Being knowledgeable is almost never enough to perform a role in medicine since no case is like the other, and human bodies are unpredictable. Therefore, errors, no matter how minimal, should be avoided or reduced, and this is what is accomplished through training [5].

Our serious game designed for Lung Cancer Radiotherapy training provides a realistic and practical platform for medical professionals and students. This game enables players to navigate through the entire clinical radiotherapy process, from initial consultations and CT scans to the final treatment stages. It is structured into ten scenes, each corresponding to a critical phase of the treatment process, thereby ensuring comprehensive coverage of all necessary skills and decision-making processes.

A Serious Game like the one we created provides a realistic medical environment with practical examples to perform on. It provides a digital platform for training where the players can fulfill the clinical radiotherapy procedure for lung cancer patients from the minute, they get a CT scan all the way to treatment for the purpose of minimizing potentially fatal errors. Our game consists of 10 scenes in total and assimilates the main 5 stages of delivering treatment to a patient.We seek to improve our project to a more advanced level and expand its use to different professionals in the field, as well as integrate more types of cancer to train on. Furthermore, our Serious Game could also be associated with a Learning Management System to record a player’s progress over the course of time.

### Expanding to Different Anatomical Sites

When considering the adaptation of our serious game model to other anatomical sites such as Head & Neck (H&N), Breast, or Pelvis, it is crucial to integrate site-specific clinical scenarios and treatment protocols. Each site presents unique challenges and requires specific knowledge and skills. For example, in designing a game for H&N, attention must be paid to the complexity of structures and the high risk of morbidity associated with treatment errors. We recommend collaborating with specialists in each field to ensure accuracy and relevance. Including common site-specific errors in the game is essential. This approach not only enhances realism but also prepares practitioners to recognize and avoid such errors in clinical practice. By simulating real-world scenarios, including common pitfalls, the game can serve as a valuable tool in improving treatment outcomes and patient safety.

### Comparison with Other Medical Serious Games

Our game enhances the educational experiences of the users by providing.

- Interactive Learning: The game’s immersive nature fosters critical thinking and decision-making skills crucial in the radiotherapy field. It mimics the real-life scenarios whithin the radiology department that medical professionals might face, enhancing thus knowledge rentention and engagement significantly [23] in their study underscore the effectiveness of such technologies in medical education, demonstrating how virtual environments can significantly enhance the educational experience by providing interactive and personalized learning interventions. This aligns perfectly with our project’s objective to utilize serious games in radiotherapy training, thereby ensuring that learners not only engage deeply with the content but also apply their learned skills effectively in real-world scenario.
- Skill Transfer and Performance: VR simulations within the game, such as those for practicing tumor contouring or radiation planning, effectively transfer skills from virtual to real-world settings, thereby improving procedural accuracy and confidence. They provide a safe environment where students can practice skills without the risk of harming a patient, essential in high-stakes fields such as surgery [24].
- Practical Applications: Specifically, in radiotherapy training, the game integrates content, simulations, and elements of gamification to provide a holistic educational experience. This is particularly useful in emergency procedures and complex treatment planning, where precision is paramount [25].
- Educational Efficacy: The game excels in rapidly disseminating complex procedural knowledge and enhancing the cognitive skills necessary for effective radiotherapy planning. This is crucial in environments where traditional training may fall short, such as during pandemics when in-person training is limited. Montalbano et al.‘s work underscores that serious games can facilitate a quicker and broader understanding of necessary safety and medical procedures than traditional educational methods, making them particularly valuable in urgent and evolving health crises like the COVID-19 pandemic [26]. This adaptability and immediacy in teaching complex procedures exemplify serious games’ potential in enhancing educational efficacy under constrained conditions.
- It significantly improves immediate knowledge acquisition as seen in domains such as oral diagnosis and treatment planning. The game’s design allows for immediate feedback and iterative learning, essential for retaining complex procedural knowledge over time. Studies like those conducted by Buajeeb et al. and Fran Valenzuela-Pascual et al. demonstrate that serious games enhance learning efficiency through dynamic feedback mechanisms that adjust to the learner’s needs, facilitating not just the acquisition but also the long-term retention of knowledge. This effectiveness is critical in healthcare education, where mastering intricate procedures and protocols can significantly impact patient outcomes [27, 28]. The integration of serious games into healthcare curricula thus supports a robust educational framework that not only improves immediate understanding but also ensures sustained knowledge retention, a vital component in healthcare training.

Serious games in radiation therapy differ from those in other medical areas primarily due to the technical nature and precision required in radiation planning and delivery. While games in other medical fields might focus on diagnostic skills or surgical techniques, radiation therapy games need to emphasize spatial reasoning, dose calculation, and a deep understanding of radiation physics [29, 30]. This distinction necessitates a unique approach to game design, focusing on these specialized skills.

### Limitations

There are many limitations that prevented this research from reaching its full potential, firstly including the length of time associated with the actual radiotherapy training process that can take months of on-field practice, thus not attaining a relatively advanced level when it comes to the serious game. Second was the independency of the game and the lack of tools available to allow the players to track their progress throughout their journey besides the results page they obtain after finishing their current game, thus depriving us from collecting an organized set of data.

## Conclusion

The integration of serious games in radiotherapy presents a promising avenue for enhancing the educational experience of new practitioners. This approach aligns with current trends in medical education, which emphasize interactive and engaging learning methods. Our review of the literature and analysis of case studies indicate that serious games can potentially shorten the learning curve for novices in radiotherapy. This effect is attributed to the immersive and interactive nature of serious games, which allows learners to acquire and apply knowledge in a safe, controlled environment.

To further validate this hypothesis, we recommend conducting empirical studies that compare the learning curves of novices trained with traditional methods versus those using serious games. Specific elements that warrant close examination include the complexity and realism of the game scenarios, the integration of real-world clinical data, and the efficacy of feedback mechanisms within these games. Such elements are crucial for ensuring the educational effectiveness of serious games in this field.

Future research should also focus on identifying the key features of serious games that most significantly impact learning outcomes in radiotherapy. This could involve qualitative studies, such as interviews or focus groups with users, to gain insights into their experiences and perceptions. Additionally, quantitative measures, like performance metrics and time-to-competency, should be employed to objectively assess the impact of these games on learning efficiency [5].

In light of these considerations, serious games emerge not only as a tool for enhancing educational engagement but also as a potential catalyst for more rapid skill acquisition in the complex field of radiotherapy [31, 32]. Our findings underscore the need for a thoughtful design and implementation of such tools, ensuring they are tailored to meet the specific learning objectives and challenges of radiotherapy training.

As future perspectives, this project needs to be benchmarked with specialists in the field of Radiotherapy, as well as medical and physics students, and technologists.

There is a lot of room to improve such a project, by creating or linking this game to a Learning Management System (e.g. e-learning system), to monitor how much the player accesses the game and how he has progressed since the first attempt.

We believe that this game is the solid base for a series of games in the radiotherapy field. It could be improved by incorporating more types of cancer. At this point, it can provide training for Healthcare Professionals entering the field of Radiotherapy. On the longer term, it is the backbone for advanced, extensive professional training.

Finally, we understand the critical importance of accuracy in medical training tools and are committed to maintaining the highest standards of clinical fidelity in our serious game. Surely, this game is first proof of concept that can be continuously validated within the medical community and the clinical information can be easily updated accordingly.

### Electronic supplementary material

Below is the link to the electronic supplementary material.

Supplementary Material 1

Supplementary Material 2

Supplementary Material 3

Supplementary Material 4

Supplementary Material 5

Supplementary Material 6

## Data Availability

The datasets used and/or analyzed during the current study are available from the corresponding author on reasonable request.

## Abbreviations

3D-CRT: 3D-Conformal Radiotherapy
CBCT: Cone Beam Computed Tomography
CT: Computed Tomography
CTV: Clinical Target Volume
DVH: Dose-Volume Histogram
GTV: Gross Target Volume
Gy: Gray unit
IMRT: Intensity Modulated Radiotherapy
Kv: KiloVolt
LaTIM: Laboratoire de Traitement de l’Information Médicale
MIP: Maximum Intensity Projection
MLC: Multi-Leaf Collimator
MV: MegaVolt
OAR: Organs At Risk
