# Supplementary material for: Serious game for radiotherapy training
Source: BMC Med Educ. 2024 Apr 26;24:463. doi: 10.1186/s12909-024-05430-1 (PMC11055359; doi:10.1186/s12909-024-05430-1)
Supplement: Supplementary file 1 — Supplementary Material 1 [file 12909_2024_5430_MOESM1_ESM.docx]

**Additional figures for Screening Simulation Scene**


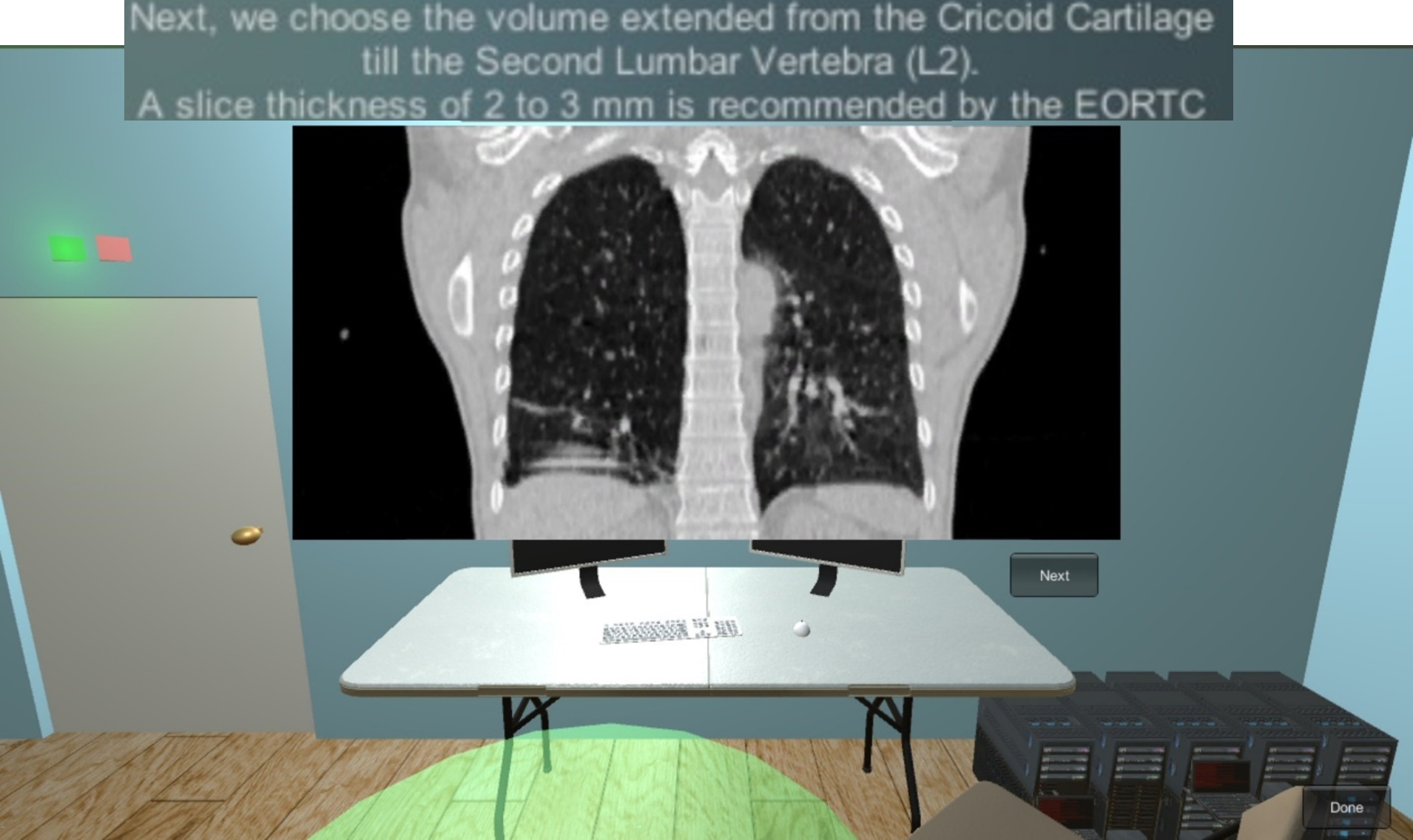


Screening Simulation Scene - CT Scan - The player is explained some standards on Thorax scanning and slice thickness requirements


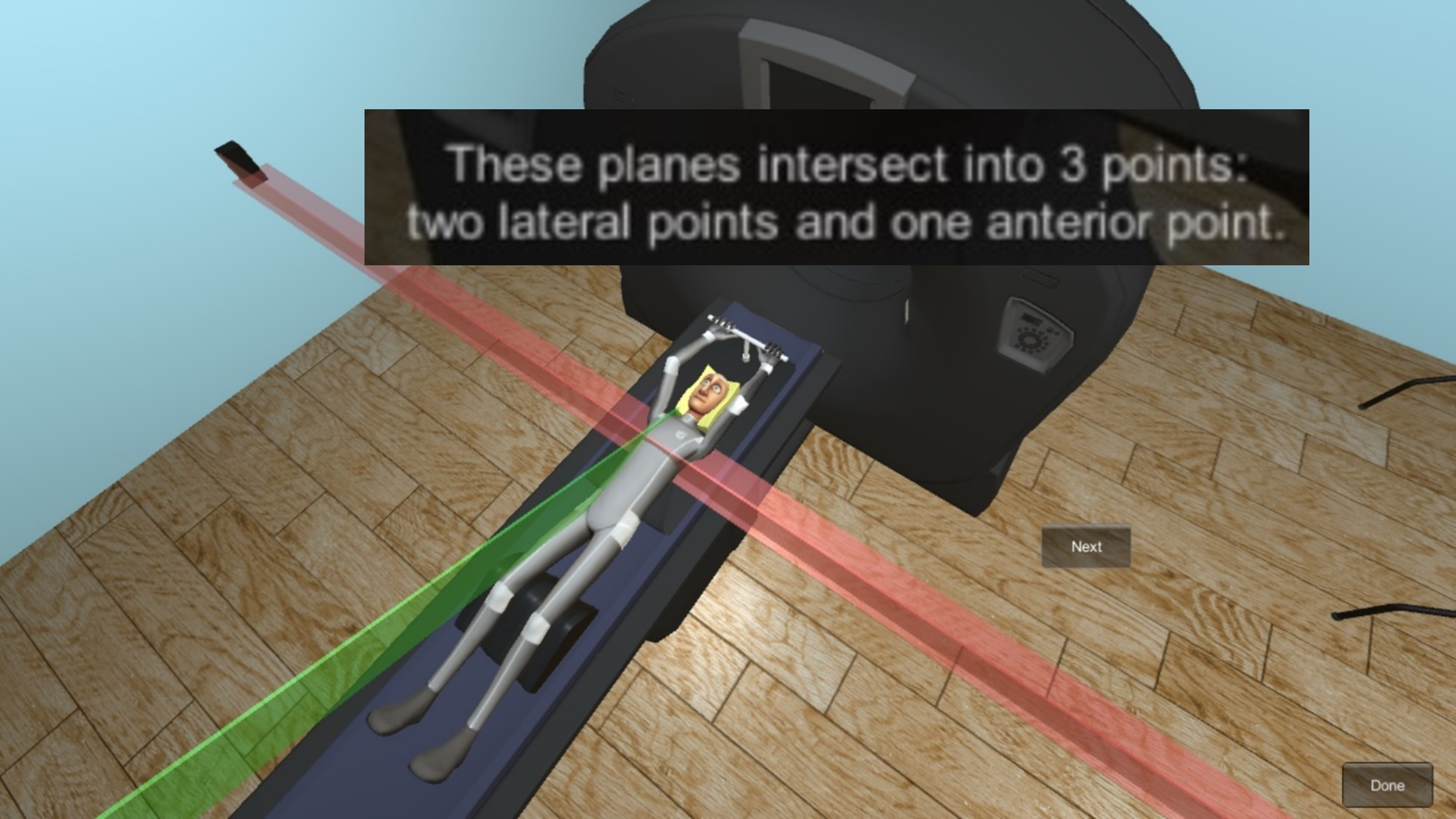


Screening Simulation Scene - Mobile Lasers – The player is explained how the mobile lasers locate the tumor and where the patient needs to be marked.
