# Supplementary material for: Serious game for radiotherapy training
Source: BMC Med Educ. 2024 Apr 26;24:463. doi: 10.1186/s12909-024-05430-1 (PMC11055359; doi:10.1186/s12909-024-05430-1)
Supplement: Supplementary file 2 — Supplementary Material 2 [file 12909_2024_5430_MOESM2_ESM.docx]

**Additional figures for Contouring Scene**


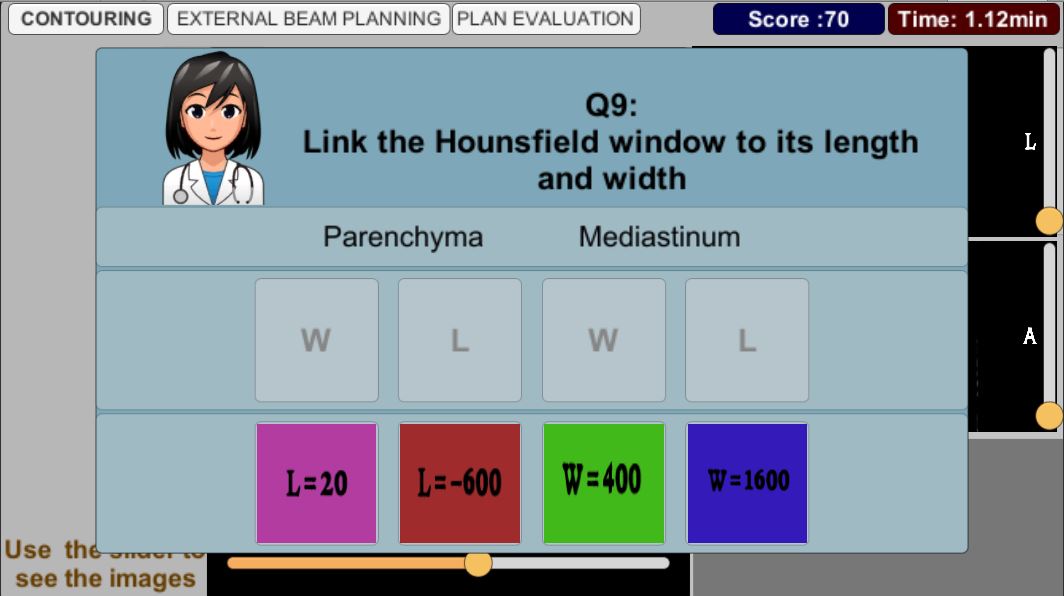


Contouring Scene - Drag and Drop Question - The first type of question is Drag and Drop, the player clicks on the desired box image and moves it with the mouse to the answer box. After moving all the boxes from the lower to the upper side, the answer is evaluated, and the scoring is decided accordingly.


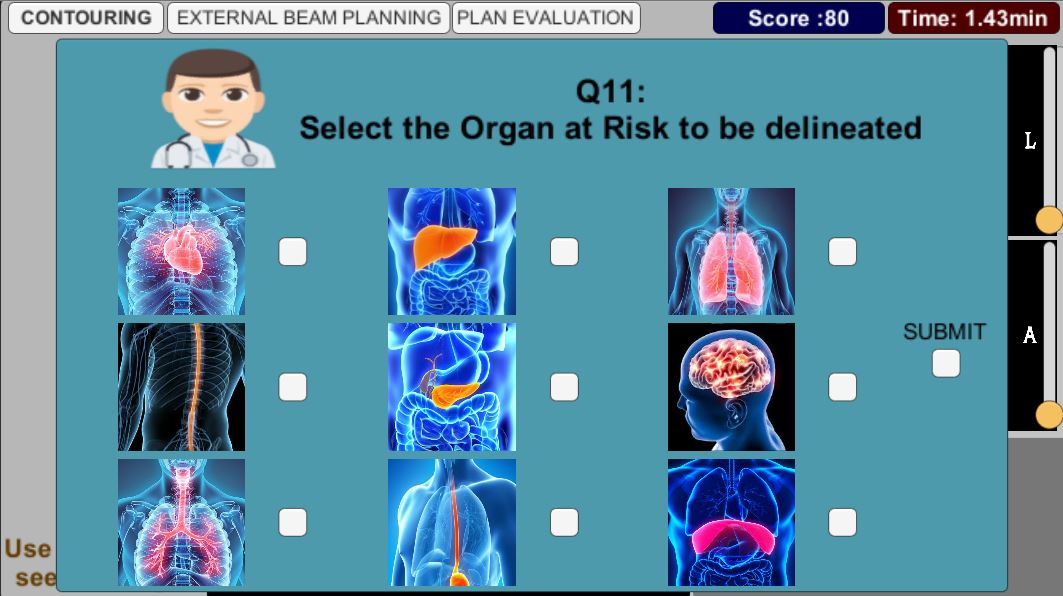


Contouring Scene - Multiple Selection Question - The player is given multiple choices to select from by clicking on the Toggle near each image. After finishing, the player submits the answer for evaluation, and moves to the next message box or question. In this question, we select the Organs At Risk to be delineated for lung cancer from nine pictures, each denoting a body organ.


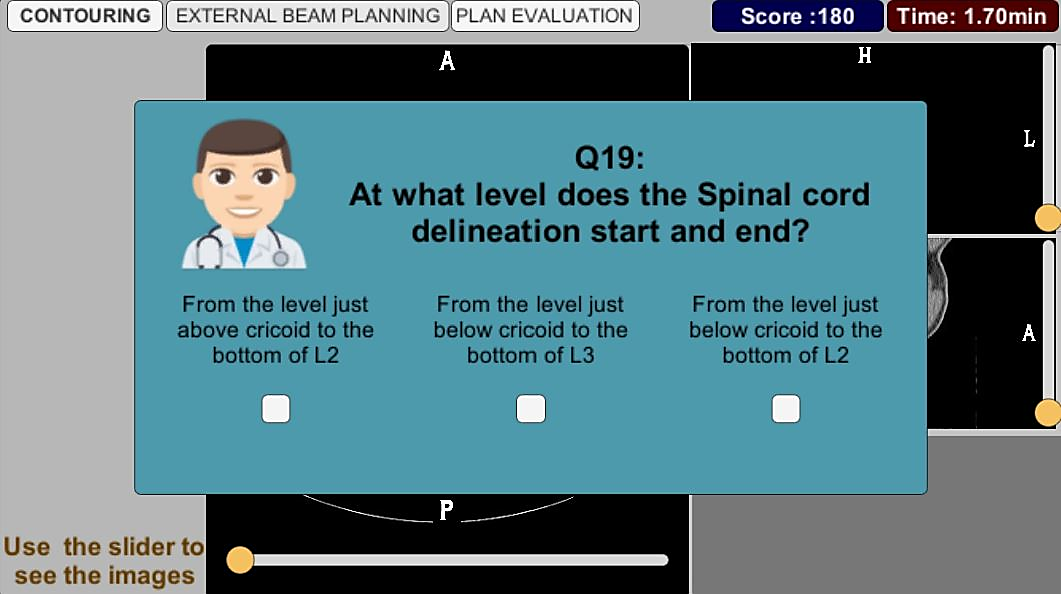


Contouring Scene - Multiple-Choice Questions - This question is based on clinical references [14]. This question is on the delineation of the Spinal Cord, and it starts just below the cricoid cartilage to the bottom of the L2 vertebra.

.
