# Supplementary material for: Serious game for radiotherapy training
Source: BMC Med Educ. 2024 Apr 26;24:463. doi: 10.1186/s12909-024-05430-1 (PMC11055359; doi:10.1186/s12909-024-05430-1)
Supplement: Supplementary file 3 — Supplementary Material 3 [file 12909_2024_5430_MOESM3_ESM.docx]

**Additional figures for External Beam Planning Scene**


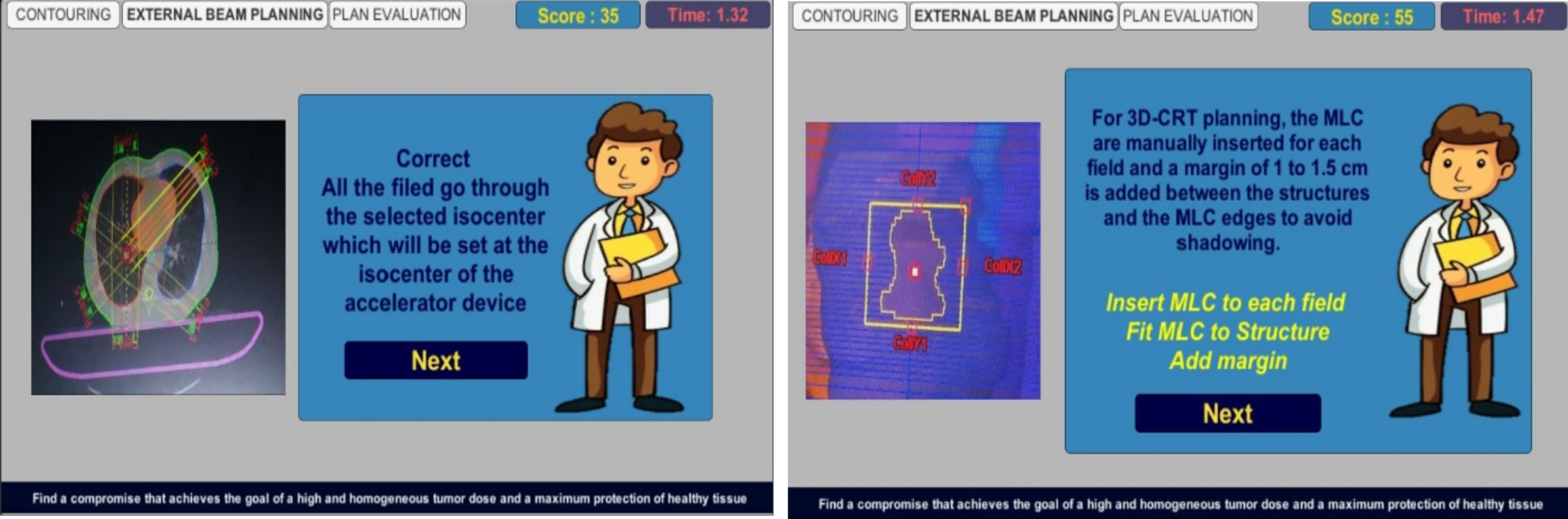


External Beam Planning Scene - Message Boxes - To interact with the player, either giving feedback or additional information..


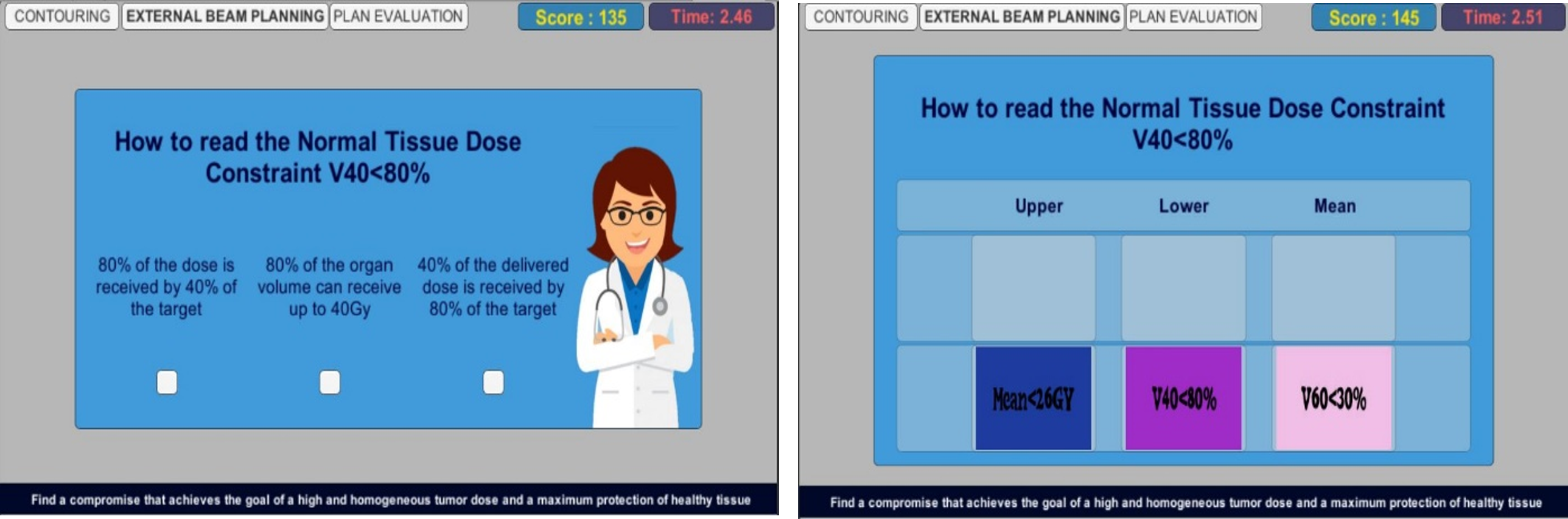


External Beam Planning Scene - IMRT Optimization Questions - Where the player needs to know how to read constraints, so he can insert them in the optimization window for a correct planning.
