# Supplementary material for: Serious game for radiotherapy training
Source: BMC Med Educ. 2024 Apr 26;24:463. doi: 10.1186/s12909-024-05430-1 (PMC11055359; doi:10.1186/s12909-024-05430-1)
Supplement: Supplementary file 4 — Supplementary Material 4 [file 12909_2024_5430_MOESM4_ESM.docx]

**Additional figure for Plan Evaluation Scene**


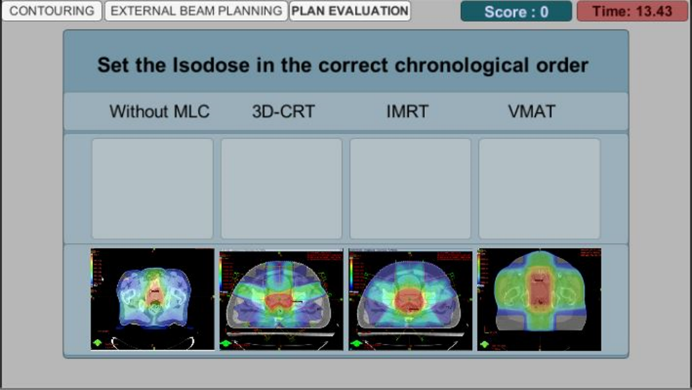


Plan Evaluation Scene - Isodose Examples - It challenges the player to guess the technology used to treat each of the cases in the images below. Before MLC's were created, the dose shape was similar to a cuboid. Afterwards, with 3D-CRT, dose shape became spherical, reducing it by 50% on the nearby organs. IMRT came to modulate the dose so it would take the exact shape of the tumor. Finally, VMAT distributes the dose into arcs, reducing hot spots near the surface.
