# Supplementary material for: Serious game for radiotherapy training
Source: BMC Med Educ. 2024 Apr 26;24:463. doi: 10.1186/s12909-024-05430-1 (PMC11055359; doi:10.1186/s12909-024-05430-1)
Supplement: Supplementary file 5 — Supplementary Material 5 [file 12909_2024_5430_MOESM5_ESM.docx]

**Additional figure for Treatment Delivery Quiz Scene**


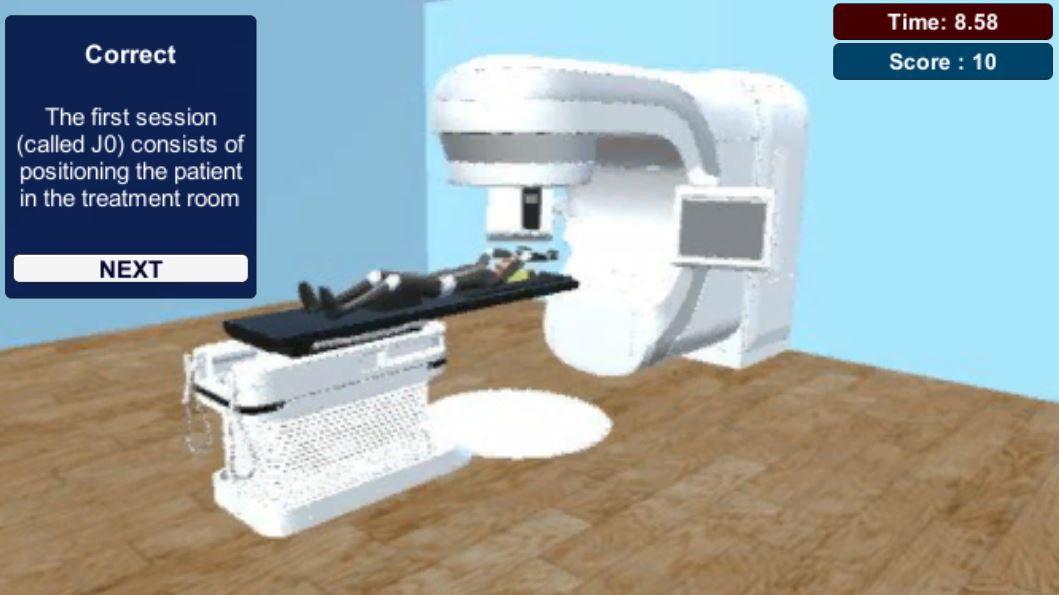


Treatment Delivery Quiz Scene - Feedback - The game provides feedback for correct or wrong answers, along with additional information to enrich the player's knowledge.
