# Supplementary material for: Serious game for radiotherapy training
Source: BMC Med Educ. 2024 Apr 26;24:463. doi: 10.1186/s12909-024-05430-1 (PMC11055359; doi:10.1186/s12909-024-05430-1)
Supplement: Supplementary file 6 — Supplementary Material 6 [file 12909_2024_5430_MOESM6_ESM.docx]

**Additional figure for Screening Quiz Scene**


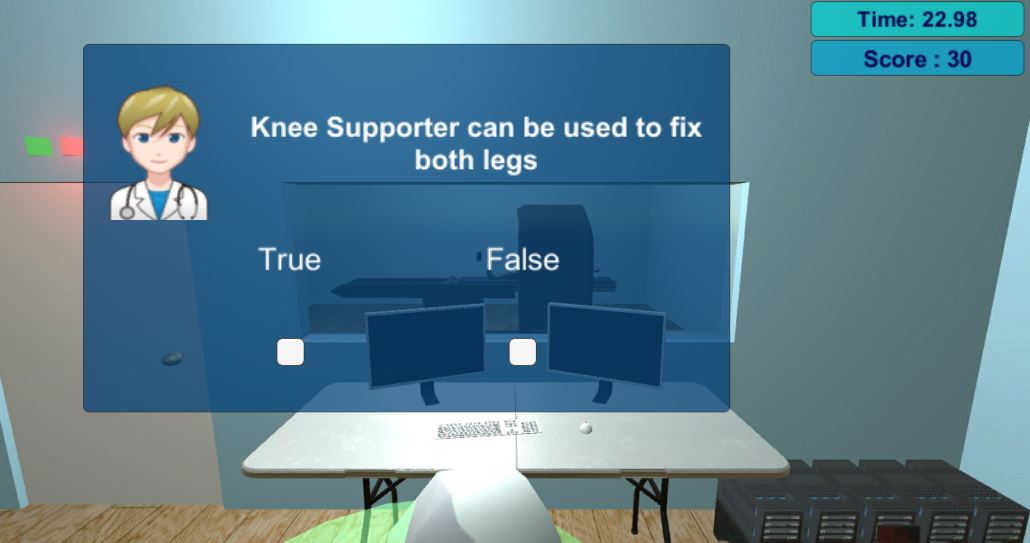


Screening Quiz Scene - True/False Question - The player selects his answer by clicking on the Toggle box below the answer. The Toggles are linked to a C# script, to check if the answer is correct, activate the audio, show the desired message box, and update the score.
